# Depressive symptom screening in elderly by passive sensing data of smartphones or smartwatches: A systematic review

**DOI:** 10.1371/journal.pone.0304845

**Published:** 2024-06-27

**Authors:** Rujira Adhibai, Hathairat Kosiyaporn, Kamolphat Markchang, Sopit Nasueb, Orratai Waleewong, Rapeepong Suphanchaimat

**Affiliations:** 1 International Health Policy Program, Ministry of Public Health, Nonthaburi, Thailand; 2 Division of Epidemiology, Department of Disease Control, Ministry of Public Health, Nonthaburi, Thailand; Helwan University Faculty of Engineering, EGYPT

## Abstract

**Background:**

The elderly is commonly susceptible to depression, the symptoms for which may overlap with natural aging or other illnesses, and therefore miss being captured by routine screening questionnaires. Passive sensing data have been promoted as a tool for depressive symptoms detection though there is still limited evidence on its usage in the elderly. Therefore, this study aims to review current knowledge on the use of passive sensing data via smartphones and smartwatches in depressive symptom screening for the elderly.

**Method:**

The search of literature was performed in PubMed, IEEE Xplore digital library, and PsycINFO. Literature investigating the use of passive sensing data to screen, monitor, and/or predict depressive symptoms in the elderly (aged 60 and above) via smartphones and/or wrist-worn wearables was included for initial screening. Studies in English from international journals published between January 2012 to September 2022 were included. The reviewed studies were further analyzed by a narrative analysis.

**Results:**

The majority of 21 included studies were conducted in Western countries with a few in Asia and Australia. Most studies adopted a cohort study design (n = 12), followed by cross-sectional design (n = 7) and a case-control design (n = 2). The most popular passive sensing data was related to sleep and physical activity using an actigraphy. Sleep characteristics, such as prolonged wakefulness after sleep onset, along with lower levels of physical activity, exhibited a significant association with depression. However, cohort studies expressed concerns regarding data quality stemming from incomplete follow-up and potential confounding effects.

**Conclusion:**

Passive sensing data, such as sleep, and physical activity parameters should be promoted for depressive symptoms detection. However, the validity, reliability, feasibility, and privacy concerns still need further exploration.

## Introduction

Major Depressive Disorder (MDD) has become increasingly prevalent in both Western and Asian elderly societies. A review suggested that the prevalence of MDD in the elderly, residing in the USA, was up to 16% [1]. It is also found that 8.7% of British elderly were diagnosed with MDD in a multicenter study [2]. In Asia, the prevalence of MDD among Indian varied from 13% to 25% [3] and the prevalence of depressive symptoms in Chinese elderly was 20% [4], respectively. The Department of Mental Health, Ministry of Public Health, reported a national prevalence of MDD among the elderly population in Thailand, estimated at 4.1% in 2008 [5]. In the most recent surveillance activity conducted in 2022, covering 68% of the Thai elderly population, the prevalence of individuals at risk for MDD was found to be only 0.4% using the “2Q” screening questionnaire (Thai version of the Patient Health Questionnaire-2; PHQ-2) and 0.03% using the “9Q” screening questionnaire (PHQ-9) [6]. However, it is noteworthy that the existing number of depressed patients was recorded at 3.5% in 2019 [7]. This incoherency in the percentage of those found to be at risk compared to the prevalence of MDD mentioned above may, in turn, reflect an ongoing pitfall of a one-time-incontiguous surveillance activity.

Furthermore, complexities in depressive symptoms screening have also been reported, attributed to the overlap between these symptoms and natural aging processes or other illnesses. This includes loss of appetite, insomnia, psychomotor retardation, and forgetfulness [8]. The elderly could also portray unique symptom presentations in comparison to other younger populations. For instance, they tend to demonstrate more somatic symptoms than emotional and motivational ones, such as having headaches and gastrointestinal pain [9, 10]. Screening questionnaires may also deliver poorer accuracy as a consequence of cognitive impairment and recall bias in the elderly [11]. Altogether, employing a self-report approach to screen for depressive symptoms in the elderly remains a persistent challenge. As a result, late-life depression may not be adequately recognized, resulting in under-diagnosis and treatment [12].

Nevertheless, technological advancement offers a new possibility for depressive symptoms screening. Passive sensing could automatically quantify moment-to-moment physiological and behavioral data via embedded sensors and connected devices, such as smartphones or wearables; regarded to as “digital phenotyping” [13]. It enables longitudinal accumulation of extensive physiological and behavioral data with minimal user interactions in a real-life setting; ensuring the objectivity, accuracy, and ecological validity of the data collected [14]. A growing body of empirical work on passive sensing data for mental health is emerging. Two systematic reviews by Mendes et al. (2022) and De Angel et al. (2022) shed light on the use of smartphones and wearables’ features as proxies of mental health status [15, 16]. The findings suggested a vigorous growth in the recent development of passive sensing applications.

Moreover, a study by Wang et al. (2018) has mapped these digital features onto MDD symptoms listed in the Diagnostic and Statistical Manual of Mental Disorders (DSM-V) which is referred to as “symptoms features” [17]. These symptom features were validated against a screening questionnaire with high validity and reliability (PHQ-8). The findings indicated a significant relationship between symptom features and depressive symptoms. For instance, longer unlock duration at study places is significantly associated with loss of interests, feelings of worthlessness, psychomotor retardation or agitation among undergraduate students [17].

Though the link between passive sensing data and depressive symptoms has been established in adolescents and adults, there is still a lack of literature in the elderly population who may be at a heightened risk of experiencing this condition. Therefore, the objective of this study is to explore the current knowledge of passive sensing data via smartphones or smartwatches to detect depressive symptoms in the elderly. This review will describe characteristics of included studies, study design, data collection tools, main outcomes presented with a focus on the association between digital features and symptom features along with related methodological limitations.

## Materials and methods

A systematic review was exercised and registered with the PROSPERO database (CRD42022341771).

### Search strategy

Search terms comprised of three themes designed to meet the topic of interest: a) elderly, b) passive sensing, and c) depression, see Table 1. Literature searches were performed across three international databases with sufficient coverage on mental health and digital health: PubMed, IEEE Xplore digital library, and PsycINFO. Hand searching was conducted on Google Scholar, supplemented by reference searching in the eligible reviewed studies, in order to enhance the comprehensiveness of the literature coverage in this review. Duplicated articles were removed prior to data selection.

**Table 1:** Search terms.

Theme	Search Terms
Elderly	elderl* OR senior* OR older OR aging OR ageing OR "old age"
	AND
Passive sensing	"screen time" OR screentime OR SMS OR text* OR call* OR accelerometer* OR pedometer* OR actigraph* OR "global positioning system" OR GPS OR sleep* OR "circadian rhythm" OR "circadian rhythms" OR sensing OR sensor OR sensors OR "digital biomarker" OR "digital biomarkers" OR "digital phenotype" OR "digital phenotypes" OR "digital phenotyping” OR "heart rate"
	AND
Depression	depress*

### Eligibility criteria

The inclusion criteria consisted of studies investigating the use of passive sensing data (behavioral and/or physiological data) to screen, monitor, track, and/or predict depressive symptoms in the elderly (aged 60 and above) via smartphones and/or wrist-worn wearables, and being published in English and in international journals between January 2012 to September 2022. Studies focusing solely on mental health patients, intervention evaluations, or studies defined depression as a confounder, were excluded from this review as same as non-empirical or unretrievable full-text studies, see Table 2.

**Table 2:** Inclusion and exclusion criteria.

Criteria	Inclusion criteria	Exclusion criteria
Population	• Elderly aged 60 or above	• Elderly who has been diagnosed or reported psychiatric symptoms of any mental disorders stated in the DSM-V
Intervention	• Studies investigating physiological and/or behavioral data derived from passive sensing either as a part or the main focus of the study • Studies that directly screen, monitor, track, or predict depressive symptoms either as a part or the main details of the study • Data collecting via smartphones and/or wrist-worn wearable devices	• Studies on intervention/treatment evaluation of any mental disorders
Comparison	-	-
Outcome	• Outcomes on the description/direct or mediated association/validity/ reliability/feasibility of passive sensing and depressive symptoms regardless of the association’s directionality	• Outcomes where depressive symptoms were found to be a confounder
Study	• Empirical research ○ Randomized controlled trials ○ Quasi-experimental studies ○ Cohort studies ○ Cross-sectional studies ○ Case-control studies ○ Qualitative studies ○ Mixed-methods studies ○ Case studies	• Non-empirical research ○ Commentaries/perspectives/opinions ○ Letters ○ Editorials ○ Protocols ○ Reviews
Others	• Studies published globally between January 2012 –September 2022	• Studies with no full-text • Grey literature • Non-English studies

### Study selection

Four researchers (HK, RA, KM, SN) were responsible for title-and-abstract and full-text screening for eligible studies. All researchers work independently to screen titles and abstracts, in which two researchers were required to make a selection consensus. A third opinion from another researcher was sought if there were any disagreements. A similar process was adopted for full-text screening to identify relevant studies related to passive sensing data and depressive symptoms.

#### Quality assessment

The quality of this review was assessed by the Joanna Briggs Institute (JBI) critical appraisal tool’s checklist for systematic review [18]. All researchers work independently to assess study quality, while HK and RA were in charge of making final decisions in response to any disagreements. The above-mentioned checklist was used as a reminder for the researchers and readers on the literature’s data quality, without having a specific cut-off score in this review.

### Data extraction

Data extraction was undertaken by a data extraction form, categorizing the information into the four main aspects as followed:

- Study characteristics: author, year of publication, setting, country, objective(s), study design, and study period.
- Methodology: target group, age, sample size, sampling technique, sample inclusion and exclusion criteria, type of device (smartphone/wearable), application(s) used, independent variables and measurement, dependent variables and measurement, confounding variables and measurement, duration of data collection per day or excluding period, and data analysis.
- Results summarized corresponding to each objective.
- Limitations and recommendations stated by the authors of each reviewed studies.

### Data analysis

The extracted data was analyzed by using a narrative synthesis. The characteristics of study were tabulated to identify their differences in settings, study designs and study periods. The study methodologies were thematized to explore target groups, sample size, data collection tools, and data analysis methods. For study outcomes, the independent, dependent and confounding variables were evaluated to find direction of relationship with of depressive symptoms and its screening performance based on statistical significance. Finally, the study’s limitations and recommendations were classified into common themes.

## Results

**Fig 1:**
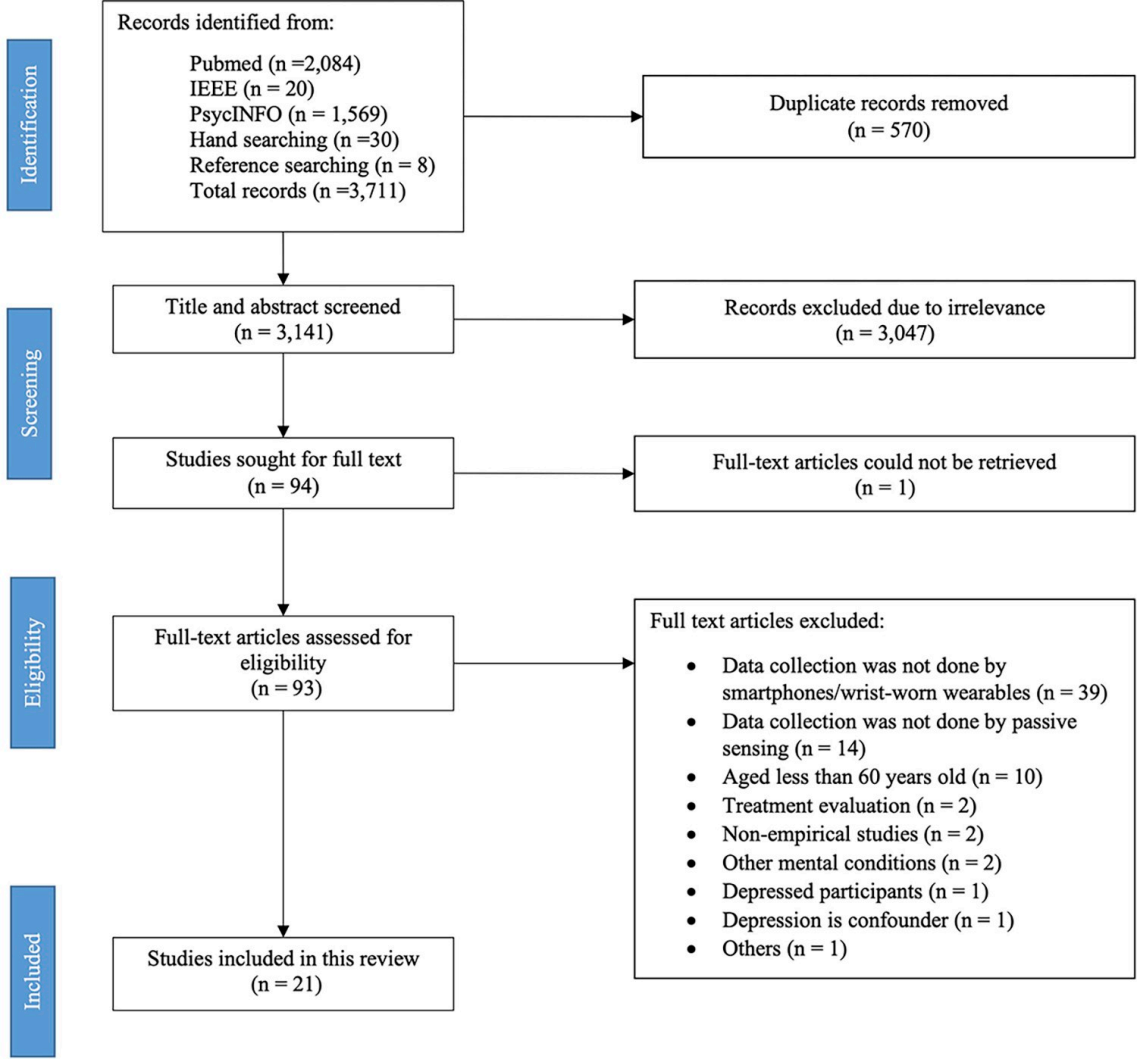
PRISMA flow of reviewed studies.

A total of 3,711 studies from all databases, hand searching, and reference searching were gathered. Then, 570 duplicated records were removed (Fig 1). Three thousand one hundred and forty-one titles and abstracts were screened to exclude irrelevant studies. Accordingly, 94 studies were sought for full text versions. One study’s full-text could not be retrieved. Hence, 93 studies were assessed for eligibility and excluded those that did not collect passive sensing data from smartphone or wearable devices, aged less than 60 years old, and others. Finally, 21 studies passed the review.

### Study characteristics

**Fig 2:**
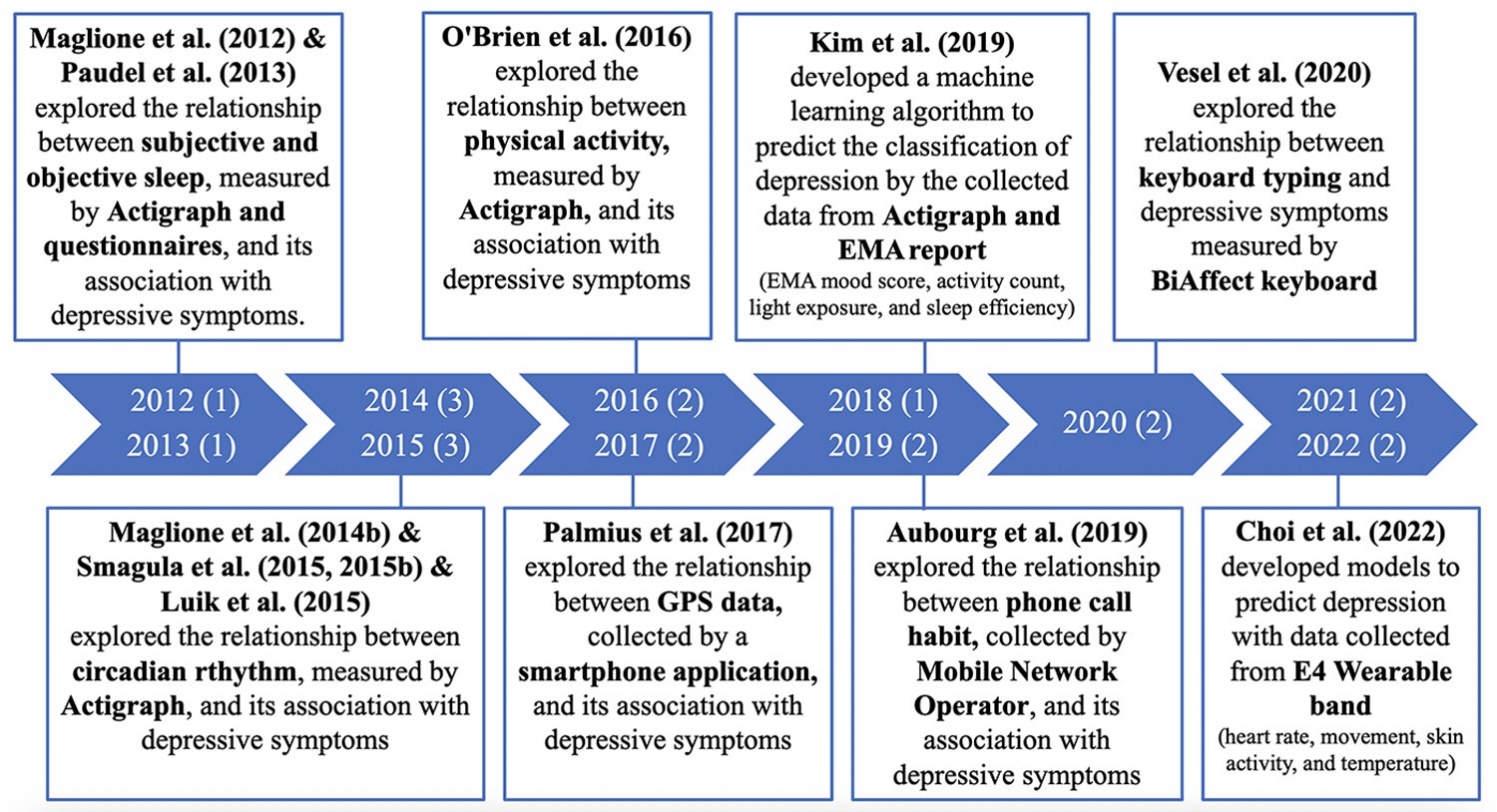
Publication year and highlighted methodology of reviewed studies. Note: Year (Number of studies); Ecological Momentary Assessment (EMA).

S1 Table summarizes the characteristics of all 21 studies, published in international peer-reviewed journals from 2012 to 2022. There has been a consistent increase in the number of studies from 2014 until the present. The trend in the evolution of these studies has shifted from analyzing a single variable to identify associations between passive sensing data; such as sleep, circadian rhythm, and physical activity, and depressive symptoms (Fig 2). Subsequently, there has been a shift in focus towards incorporating multiple parameters to develop models predicting depression. This involved exploring additional passive sensing data, such as typing patterns, phone call habits, and biometric parameters. The majority of these studies (n = 16) were conducted in Western countries, including North America (n = 9) [19–27] and Europe (n = 7) [28–34], while the rest were conducted in Asia (n = 3) [35–37] and Australia (n = 2) [38, 39]. The subjects of 11 studies were from community settings [20, 21, 23, 26–28, 30, 32–34, 37] and eight studies were selected from facility-based centers [19, 22, 24, 25, 31, 36, 38, 39]. Only the sample of one study was from social and welfare center [35] and one did not identify the setting type [29].

Regarding study design, most studies (n = 12) adopted a cohort design [19, 20, 22, 24, 26, 28–31, 34–36], followed by a cross-sectional design (n = 7) [21, 23, 25, 27, 32, 33, 37] and a case-control design (n = 2) [38, 39], respectively. The objectives of the studies were mostly focused on the measurement of passive sensing data, such as physical activity and sleep. They aim to explore the association between these data and psychological disorders, particularly mood disorders. Additionally, confounding effects of demographic factors was also addressed against passive sensing data and psychological symptoms.

### Data collection

**Fig 3:**
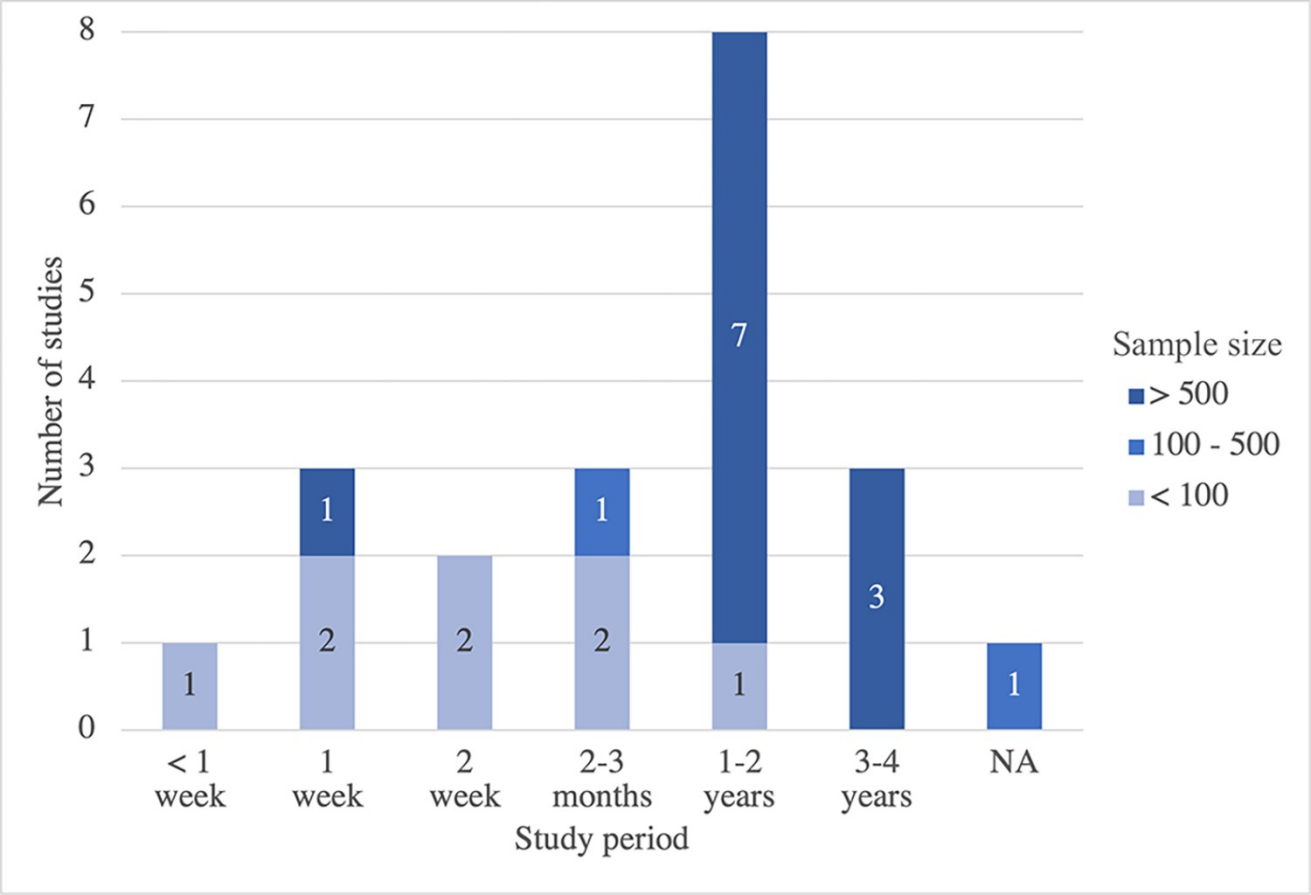
Sample size and study period of reviewed studies.

According to S2 Table, nearly all studies in this review targeted an elderly population aged 60 years and older, with an average age ranging from 63.8 to 83.6 years old (total age range from 60 to 94 years old). However, a few studies had a mixed age range under and over 60, accounting for mean ages of 37.8 (standard deviation (SD) = 12.3) to 46.0 (SD = 14.0), respectively [20, 30]. The study period ranged prominently from 1 to 2 years, with a large number of samples ranging from 805 [27] to 3,020 [23] (Fig 3). The study design was primarily composed of continuous cross-sectional studies. In contrast, cohort and case-control studies mostly had a shorter study period and smaller sample size, typically less than 150. Only five cohort studies had a higher number of participants, ranging between 397 and 2,933 [19, 20, 22, 24, 26].

Overall, the inclusion criteria for the cohort studies were mostly specific to age, lack of historical or current neurological diseases, psychiatric conditions from medical diagnosis or screening test, and consent to participate. Conversely, the exclusion criteria included those who had known or significant cognitive impairment, severe or unstable physical illness or problems related to physical movement (such as brain injury, stroke, diabetes mellitus), and a recent history or current evidence of substance abuse. In case-control studies, all the cases were identified as patients while the controls were healthy individuals [38, 39].

### Data measurement and analysis

Among 21 studies, nine studies investigated the role of sleep as an independent variable; six focused on objective and subjective sleep [21, 24–26, 33, 39] and three focused on only objective sleep [23, 32, 36], see S3 Table. The most widely used measurement tool for objective sleep was wrist actigraphy (wearable devices); tracking various parameters, such as sleep duration and efficiency. For subjective sleep, the Pittsburgh Sleep Quality Index (PSQI) was the most commonly used questionnaire (n = 5) [24–26, 33, 39], followed by the Epworth Sleepiness Scale (ESS) (n = 2) [21, 25]. Independent variables apart from sleep were physical activity (n = 8) [27, 29, 31, 32, 34–37], circadian rhythm (n = 5) [19, 22, 23, 33, 39], daily step counts (n = 1) [27], GPS (n = 1) [30], phone call records (n = 1) [28], heart rate, skin activity and temperature statistic features (n = 1) [35], mood ratings (n = 1) [20], and depression (n = 1) [38].

Passive sensing data were collected from either wearable device in various brands, such as Actiwatch, Empatica E4, SleepWatch-O, and movisens3, or smartphones through network communication operator or application, such as BiAffect keyboard. The sensitivity and specificity of actigraphy from wearable devices were reliable in detecting sleep and physical activity patterns [22, 24, 36] while it had not been addressed in the smartphone. For data collection from wrist-worn devices, the user protocol suggested that adequate time period for data collection ranged from 10 to 16 hours a day for at least three to 14 consecutive days to ensure data validity. Some studies preferred participants to wear these devices on non-dominant wrist and allowed them to take off these devices when they worked with water, slept, or recharged the band.

Most studies (n = 17) had depression as either the sole or one of their dependent variables, and some utilized two depressive symptom measurements, see S3 Table. The most commonly used questionnaires was the Ten-item or Fifteen-item Geriatric Depression Scale (GDS-10 or GDS-15; n = 11) [19, 22–26, 28, 31, 32, 37, 39], Korean Short Geriatric Depression Scale (SGDS-K; n = 2) [35, 36], Nine-item Patient Health Questionnaire (PHQ-9; n = 2) [27, 35], and the Center for Epidemiologic Studies-Depression scale (CES-D; n = 2) [21, 33]. Other dependent variables included apathy (n = 1) [29], sleep (n = 1) [38], keyboard typing features (n = 1) [20], mental well-being (loneliness, happiness, and global mental health; n = 1) [32]. Demographics, along with other variables, such as comorbidity, medication used, alcohol consumption/smoking/drug used/caffeine used, body mass index, activity of daily living, and cognitive function, were collected as confounders in 18 studies, see S3 Table [19–28, 30–34, 37–39].

Various statistical analysis techniques were applied, such as chi-square, Mann-Whitney U t-test, and Kruskal Wallis ANOVA tests for univariable analysis, see S3 Table. For multivariable analysis, regression analyzes (n = 11) were found to be the most frequent means of statistical analysis [21, 23, 25–27, 30–32, 36–38]. The most frequently used types of regression model were logistic regression. Some studies employed machine learning model, such as random forest and K-mean techniques [30, 35, 36].

#### Study outcomes

**Fig 4:**
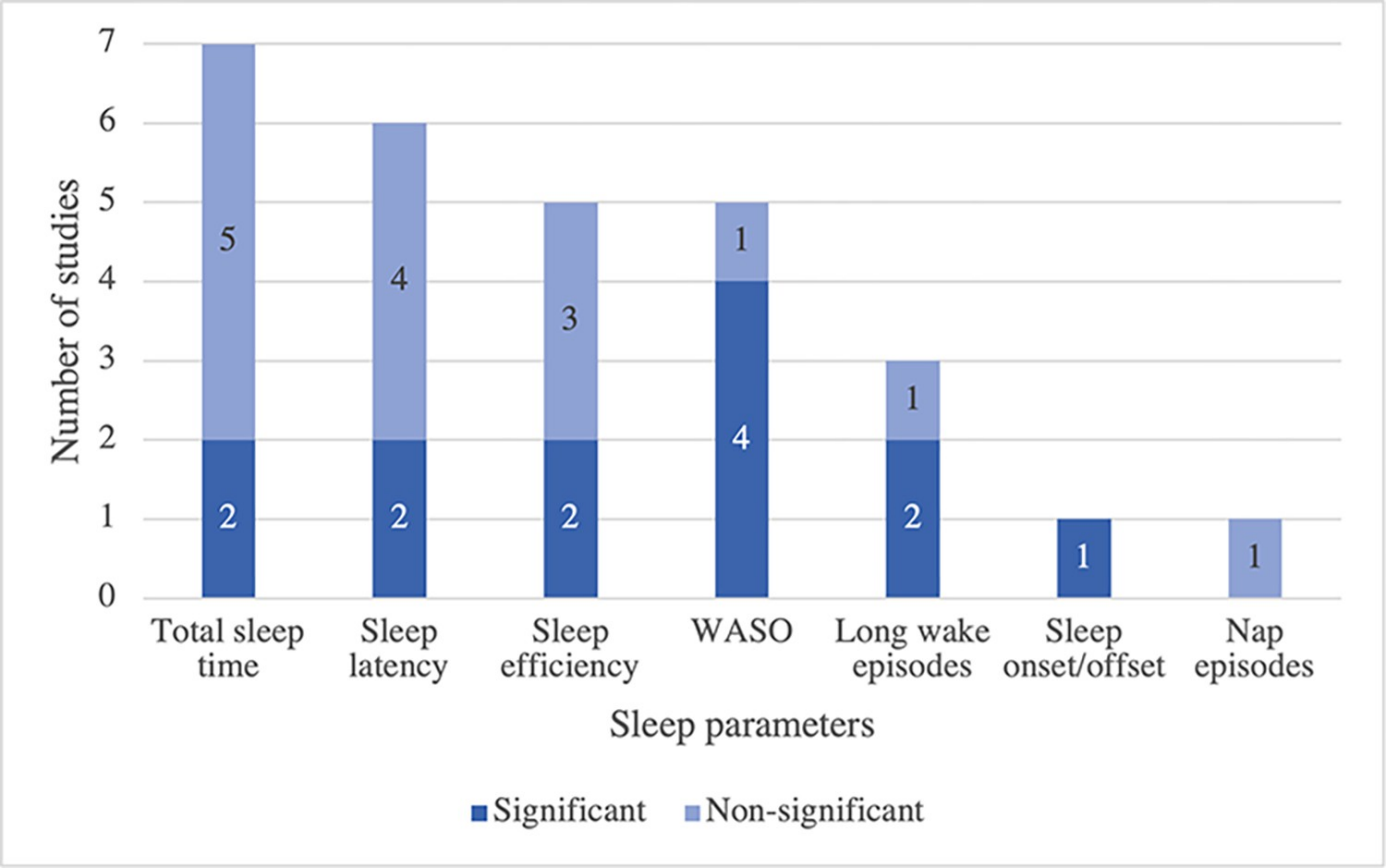
The association between sleep and depressive symptoms of reviewed studies. Note: Total sleep time (TST) referred to minutes sleep between bed time and wake time; Sleep latency referred to amount of time from being fully awake to sleeping; Sleep efficiency referred to amount of sleep minutes divided by minutes in bed; Wake after sleep onset time (WASO) referred to amount of awake minutes after sleep onset occurs; Long wake episodes referred to number of wake episodes between sleep onset and wake time exceeding 5 minutes; Sleep onset/offset referred to the time at which sleep onset happens and individual awakens; Nap episodes referred to number of inactive episodes between wake time and sleep onset exceeding 5 minutes; significant mean statistically significant at 95% confidence level.

Regarding the influence of sleep on depression, several studies revealed a significant relationship between actigraphically-assessed sleep and depressive symptoms in the elderly, see Fig 4 and S3 Table. There were various parameters of sleep duration and characteristics, such as nighttime wakefulness. For sleep duration, the association with depressive symptoms was inconsistent across studies; for example, a study by Paudel et al. (2013) suggested that total sleep time was not related to greater odds of depression at follow-up parameters [26], while a study by Alcántara et al. (2016) revealed that depression was associated with short sleep duration (adjusted prevalence ratio (PR) = 1.47; 95% confidence interval (CI) = 1.11–1.94) [21].

Regarding sleep characteristics, several studies have examined their association with depressive symptoms. Wakefulness after sleep onset (WASO) was found to be related to depressive symptoms in three studies, with a potential exacerbating effect over time when the duration of WASO exceeded one hour [24, 25, 33]. Other sleep characteristics, such as sleep efficiency, sleep latency, and long wake episodes, yielded mixed results in terms of statistical significance. For example, a study by Paudel et al. (2013) demonstrated in age- and site-adjusted models that reduced sleep efficiency (odds ratio (OR) = 1.88; 95% CI = 1.13–3.13), prolonged sleep latency (OR = 1.77; 95% CI = 1.04–3.00) and multiple long-wake episodes (OR = 1.69; 95% CI = 1.15–2.47) were associated with increased odds of depression at follow-up [26]. However, these associations lost statistical significance after adjusting for the number of depressive symptoms at baseline [26]. For circadian rhythm measurement, a study by Maglione et al. (2014b), Pye et al. (2021), Luik et al. (2015), and Smagula et al. (2015a and 2015b) showed that the decrease in robustness and amplitude, along with increased fragmentation of the circadian rhythm related to greater level of depressive symptoms [19, 22, 23, 33, 39].

**Fig 5:**
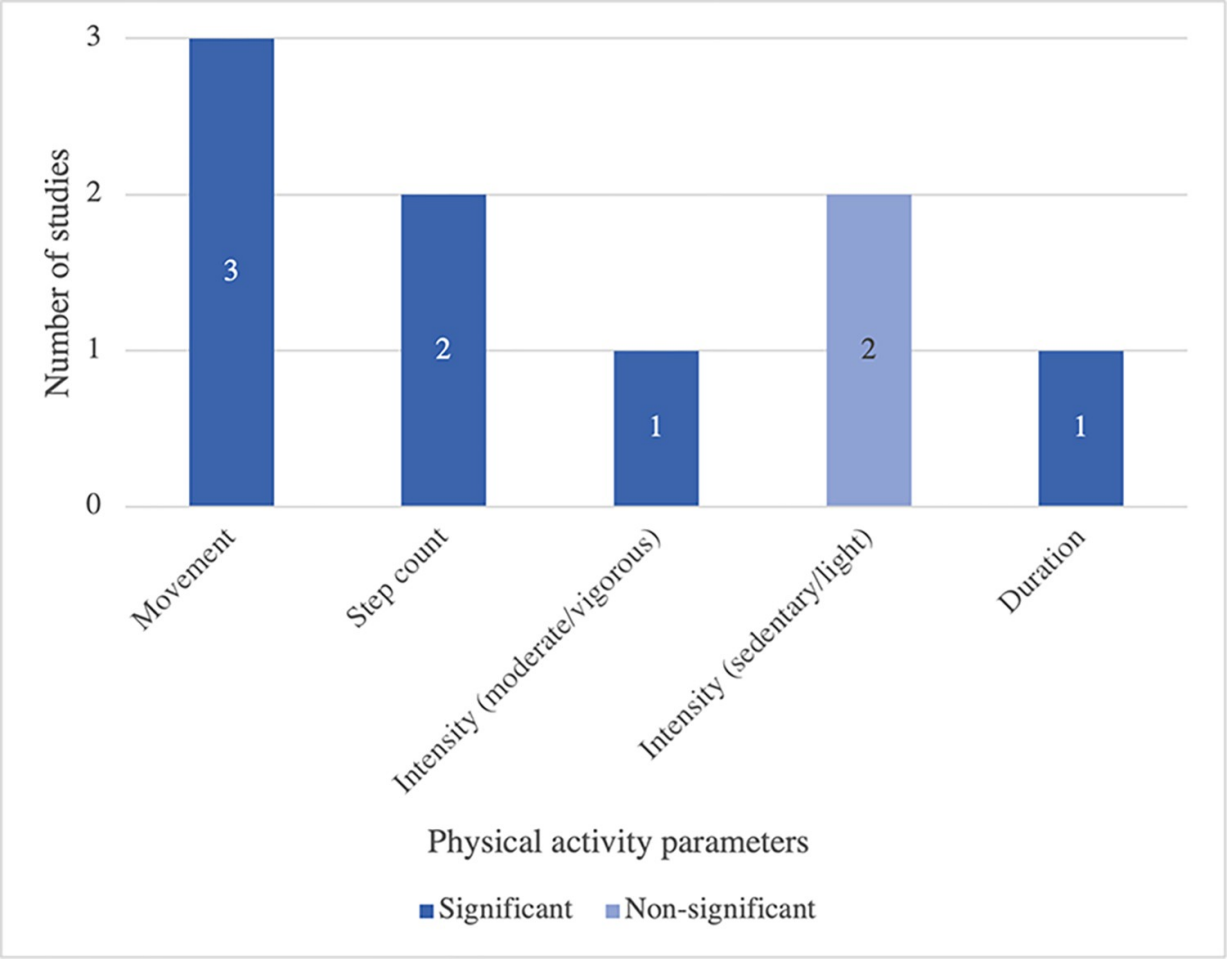
The association between physical activity and depressive symptoms of reviewed studies. Note: Movement referred to activity count, entropy, jerk; significant mean statistically significant at 95% confidence level.

A common pattern of relationship between physical activity and depression was depicted across related studies, showing a significant relationship between physical activity and depressive symptoms (Fig 5). Physical activity in a form of movement data was considerably lower (t = 3.63, p = 0.001) in elderly with depression when compared to the control group, especially in the morning [31]. Another study evidenced that daily depressive mood was negatively associated with within-person daily physical activity, an increase of one unit in physical activity (unit of standardized daily step count) was associated with a 14 percent decrease of daily depressive mood within-person [34].

**Fig 6:**
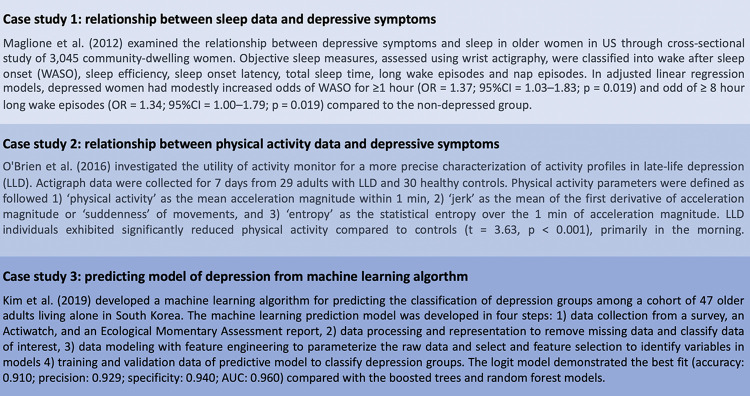
The case study of reviewed studies.

Some studies found an association between passive sensor data and depression. To elaborate, more severe depression was related to more variable typing speed (p < 0.001), shorter session duration (p < 0.001), lower accuracy (p < 0.05) [20], and was associated with reactive phone call users (those who phoned less than responding to incoming phone calls) compared to proactive phone call users (those who phoned more than responding to incoming phone calls) [28]. Few studies analyzed passive sensor data with depression in machine learning model showing that the 4-Multi-layer Perceptron of heart rate, accelerometer, skin activity and temperature had 69% accuracy and 78% recall [35], and the one analyzing five features of geographical location had 85±1.6% accuracy, 84±1.4% recall, 87±4.7% specificity, and 86±2.2% F1 score [30]. The case studies of this review were selected to highlight the methodologies and results obtained, see Fig 6.

Nonetheless, there were still some shared limitations among all these studies. First, it was dominantly mentioned that most studies have limited generalizability to the larger population due to their small sample sizes [28, 29, 33] and restricted sample characteristics [22–25, 27, 36, 38]. Second, in almost all cross-sectional studies, causality and directionality could not be concluded due to the nature of the study design [21, 23, 31, 33, 37, 38]. Third, a few studies stated that they had a short period of follow-up [22, 26, 32], and failed to eliminate residual confounding factors [19, 22, 32].

Certain studies faced limitations in attaining gold standard measurements. Among all studies on sleep, it is notable that unlike the use of polysomnography (PSG), actigraphy is not the gold standard assessment of sleep despite being noninvasive and has been validated across different demographics [24, 25]. Similarly, a clinical diagnostic interview was also not implemented as a gold standard to evaluate depressive symptoms; hindering the ability to make inferences on diagnosis [21, 23–26, 32, 35, 36].

A considerable number of studies provided recommendations for future research. First, future research should incorporate a bigger sample size and more diverse sample characteristics, such as a multi-ethnic cohort to ensure the generalizability of the findings [21, 27, 28, 34, 39]. Second, longitudinal or prospective cohort studies should be carried out to further examine the development, temporality, and prodrome signs of the relationship between the variable of interest and depression [21, 26, 27, 32] as well as provide a longer assessment or follow-up period [34]. Lastly, these recommendations shed additional light on how passive sensing data could be used as an early identification of depressive episodes, guiding future self-management plans and preventive measures [30].

#### Quality assessment

Table 3 and S4 Table summarizes the quality assessment of reviewed studies. The quality assessment of cross-sectional and case-control studies showed that all reviewed studies had low risk of bias. Only two cross-sectional studies had not clearly defined the inclusion criteria [33, 37] and one of case-control studies had not addressed strategy in dealing with confounding factors [38]. On the other hand, cohort studies had higher risk of bias. Most of them revealed common pitfalls on the incompleteness of follow-ups, strategies to deal with confounding effects, and unclarity whether participants were free of outcomes at the beginning of study. Half of all studies did not justify their reasons for loss to follow-up (n = 6) [20, 24, 28–30, 34]. Although reasons for loss to follow-up were mentioned in some studies, they neither utilized strategies to deal with incomplete follow-up nor stated its demographics, such as characteristic description (n = 10) [19, 20, 22, 24, 28–30, 34–36]. Confounding factors were also not explicitly stated in some studies (n = 4) [28, 29, 35, 36] and some did not have strategies to deal with confounding factors such as adjustments in data analysis (n = 7) [20, 26, 28–30, 35, 36]. In addition, the follow time was inadequate to ensure occurrence of outcomes (n = 3) [29, 31, 34], unsure whether participants were free from diseases at the beginning (n = 7) [19, 20, 22, 28–30, 35] and statistical methods were inappropriate in cohort studies (n = 4) [20, 28, 35, 36].

**Table 3:** Quality assessment score of reviewed studies.

Study design	Author	Total score (%)
Cohort study	Choi et al. (2022)	54.5
	Smagula et al. (2015a)	81.8
	Vesel et al. (2020)	54.5
	Kim et al.(2019)	63.6
	Aubourg et al. (2019)	36.4
	O’Brien et al. (2016)	81.8
	Maglione et al. (2014a)	81.8
	Palmius et al. (2017)	63.6
	Gruenenfelder-Steiger et al. (2017)	72.7
	Abbas et al. (2022)	45.5
	Paudel et al. (2013)	90.9
	Smagula et al. (2015b)	81.8
Cross-sectional study	Cabanas-Sánchez et al. (2021)	100.0
	Alcántara et al. (2016)	100.0
	Luik et al. (2015)	85.7
	Maglione et al. (2012)	100.0
	Maglione et al. (2014b)	100.0
	Lee et al. (2014)	100.0
	Asai et al. (2018)	85.7
Case-control study	Pye et al. (2021)	100.0
	Hoyos et al. (2020)	90.0

Note: exclude NA from total scoring

## Discussion

The discussion of this review is aligned with the results, which were classified into four parts: a) study characteristics, b) passive sensing data and depressive symptoms, c) quality assessment and d) study limitations and recommendations.

### Study characteristics

The reviewed studies were conducted across diverse settings, which expanded over time. A systematic review by de Angel et al., (2022) about digital health tools for the passive monitoring of depression in the general population revealed a growing number of studies since 2013 [16]. However, studies conducted in the elderly are not as prevalent as those in adults or adolescents; accounting for only five studies out of 52 studies [16]. Hence, studies on passive sensing and depression should be promoted in the elderly population.

Moreover, studies in the elderly were mostly conducted in High-Income Countries and populated in community-based settings. According to the Connectivity in the Least Developed Countries Status report 2021 by International Telecommunication Union, there were 260 million Internet users in the least developed countries in 2020, almost twice of users in 2016, representing about half the world average penetration (51% in 2019) [40]. Consequently, it should be noted that none of these studies were conducted in African, South American, South, and Southeast Asian regions despite having a substantial increase in mobile phone and internet access in these areas. In turn, studies in these regions, especially in the developing countries, need to be supported to bridge the existing research gap and enhance the understanding of passive monitoring tools in diverse cultural and socio-economic contexts.

### Sleep and depressive symptoms

Many aspects of sleep were gauged by wrist-worn actigraphy to investigate its association with depressive symptoms in the elderly population. Key findings from this review suggested that some sleep parameters appear to be promising proxies of depressive symptoms; especially sleep characteristic variables, such as WASO, and circadian rhythm variables [19, 22–25, 33, 39].

WASO were found to be related to depressive symptoms, which may aggravate the symptoms progressively if it persists for an hour or longer [24, 25, 33]. Consistent with previous research, depressed patients reported having more sleep disturbance as evidenced by a greater degree of reported WASO and sleep efficiency as compared to healthy individuals [41]. This is also supported by the findings from both community and clinical settings that sleep disturbance was found to be a risk factor for the incidence and recurrence of depression in the elderly [42, 43].

Two studies investigated circadian rhythm and depressive symptoms. Circadian rhythm is defined as a 24-hour pattern in physiology and behavior, controlled by the suprachiasmatic nuclei and plays a key role in sleep/wake cycle regulation [44, 45]. A study by Li et al., (2013) shed light on the link between these two variables, suggesting that depressed patients had significantly disrupted circadian rhythm [46]. Current evidence suggested depression has been linked to genes responsible for the generation and regulation of circadian rhythm [47]. Since the underlying mechanisms of circadian rhythm and sleep are interrelated, it could be auspicious to gauge both variables simultaneously to broaden current knowledge on their combined influences on depression.

However, it is important to note that not all sleep parameters were found to be related to depressive symptoms in the elderly across all studies. To elaborate, there was no association between TST and greater risks of depression at follow-up in a large cohort of community-dwelling older men [26]. Inconsistent with some research, a reduction in TST was evidenced in depressed patients and it is especially apparent in older depressed patients [21, 32]. It is expected that lower TST would be reported since insomnia is one of the common depressive symptoms among this population [9]. In addition, the association between other sleep parameters, specifically sleep efficiency and sleep latency, and depression has shown inconsistency in the literature. Depression disrupts the regulation of the sleep/wake cycle, which subsequently impacts sleep continuity measures such as sleep efficiency and sleep latency [44]. Therefore, the extent to which TST, sleep latency, and sleep efficiency in specific contexts contribute to the development of depressive symptoms remains to be elucidated. Further research, employing rigorous explanatory studies, is needed to identify a potential causal relationship.

### Physical activity and depressive symptoms

Another variable that was closely examined alongside depressive symptoms in the elderly was accelerometer-derived physical activity. Overall, the findings suggested that there is a negative relationship between physical activity and depressive symptoms. In line with previous research, depressed patients aged 60 and above were more likely to adopt a sedentary lifestyle [48] and were less physically active than their non-depressed counterparts [49]. Recent studies suggested that depressive symptoms may restrict the elderly from performing their regular physical activities [50], contributing to the deterioration of physical function and leading to an overall decline in physical activity [51]. Hence, lower physical activity could potentially be interpreted as a possible hallmark of depressive symptoms.

This review also shed additional light on the pattern of physical activity that is beneficially associated with depression, revealing that moderate-to-vigorous physical activity (MVPA) could reduce depression symptoms [32]. The findings are consistent with previous randomized-controlled trials found that three sessions of walking per week at either moderate or vigorous-intensity could lessen depressive symptoms in older adults with insomnia [52]. Furthermore, it is necessary to explore various parameters in the measurement of physical activity. Movement data, such as accelerometer count or step count, which can be easily collected using everyday devices, has shown promise as a favorable indicator that is significantly associated with depression in numerous studies [27, 29, 34, 36]. Therefore, physical activity parameters can be proxies for depression in elderly.

### Other variables and depressive symptoms

Some variables and their relationships with depressive symptoms were being less explored than others. There is a mix of positive and negative association between these factors and depression; highlighting that the combination of multiple variables could provide more accuracy for detection. The details of each variable are as follows:

Only a few studies explored the relationship between heart rate and depressive symptoms in the elderly. Recent research has shed light on autonomic dysfunction changes due to depression, with declined heart rate variability (HRV) and cardiac parasympathetic activity [53–55]. Agelink et al., (2001) revealed that parasympathetic HRV indices are inversely related to depression severity [53]. However, an epidemiologic study by Krishnan et al., (2004) conducted in middle-aged and elderly participants, did not discover an association between HRV and depression [56] likewise with the findings from this review. Thus, this variable should be studied in conjunction with other factors and there might be rooms for future research to explore such associations in specific settings.

Smartphone-related parameters such as Global Positioning System (GPS), phone calls or typing patterns have not been widely investigated in relation to depressive symptoms in this population. Though little is known about the link between GPS and depressive symptoms in the elderly, the relationship was evidenced in the younger demographics [17, 57]. A study in Korea proposed that cell phone utilization could reflect social interaction and social support in elders who lived alone [58]. The findings suggested that those who do not own a cell phone possessed a greater risk of depression than those who utilized text messages and phone calls and even those who only browsed the internet [58]. Moreover, a study about typing pattern and depression conducted in a younger group showed appropriate sensitivity and specificity of touch screen typing analysis model (sensitivity 82%; specificity 86%) [59]. It potentially explained the lack of social function and psychomotor retardation is a common component of depression [60]. Future research is needed to explore the relationship between smartphone-related parameters that reflect psychomotor symptoms in depression within this age group.

Only few studies evaluated the screening performance in terms of sensitivity, specificity, and predictive value (positive and negative). The recall and specificity serve as metrics for tool performance evaluation to detect positive and negative results among cases and non-cases, respectively. From this review, the recall and specificity of multiple sensors, heart rate and GPS were acceptable because it was higher than 70% [30, 35]. In addition, the predictive value, such as accuracy, is used to evaluate predictive performance of tools. However, the predictive model of GPS and heart rate were higher than 70% except multiple sensors of heart rate, accelerometer, skin activity, and temperature [30, 35]. Therefore, there is potential for the application of passive sensing data to detect depressive symptoms with appropriate level of screening and predictive performance; nevertheless, further studies that focus on these analyzes is still necessary.

### Quality assessment

The overall quality of all studies was acceptable, with the exception of some cohort studies that have higher risk of bias compared to other study designs. There are some key worth mentioning points as followed.

First, most of the study are cohort studies, followed by cross-sectional and case-control studies. Although a cohort study is appropriate for identifying associations between passive sensing data and depression due to utilizing a longer period of study, a more rigorous design for causal inference such as randomized control trials should be promoted. When applying quality assessment for cohort studies, most did not declare loss to follow-up reasons; therefore, the loss to follow-up should be better handled in future cohort studies.

Second, the sampling method, recruitment criteria, and sample size are essential to ensure the generalizability of the findings. In cohort studies, the sample size is smaller than cross-sectional studies. Furthermore, some studies highly limited the inclusion characteristics of the elderly, such as only having either gender, Caucasian race, those aged older than 80 years old, or only studying in certain countries. Therefore, the studies in various group of elderly should be expanded to ensure generalizability.

Third, the measurement for exposure and outcome variables still lacks standardization. Using clinical interviews for depression detection and standardized passive sensor detection such as PSG for sleep, are proposed to classify participants, confirm diagnosis, and capture actual behavioral data. Recent studies discussed that PSG assessments could be costly and lengthy due to their laboratory settings: inflicting unfamiliar sleeping environments on the participants and undermining the findings’ ecological validity [41]. Though actigraphy is not the gold standard measurement, it is still argued to be an unobtrusive measure of sleep-wake disturbances [41] and possess moderate level of overall agreement (69.4%; K = 0.386, p < 0.05) in sleep-wake state identification with PSG [61]. It also has become a preferred option over self-report measures for the older demographics [62]. From a practical point of view, actigraphy seems to be an acceptable means of sleep assessment that could offset laboratory-grade accuracy but the validity and reliability should be further explored.

Finally, confounding factors have to be identified, including factors that can influence passive sensing data and depressive symptoms. Demographic factors, such as age and gender, medical history, medicines used, and degree of daily activity, can affect passive sensing data and depressive symptom detection. Therefore, all these variables and corresponding strategies to deal with them should be identified and adjusted to ensure accurate result interpretation.

### Limitations and future research

There are still some limitations to this review. First, this systematic review derived relevant studies from only three databases, whereby only one focuses on computer science (IEEE). Second, other relevant studies, such as grey literature or conferences proceedings were omitted. Besides, only peer-reviewed studies published in English were included in this review due to the limited time and resource to seek translation by the research team. Future reviews could take in various types of literature and integrate other computing machinery and/or electronic engineering databases to broaden literature coverage and ensure the findings’ richness. Third, this review did not analyze other aspects or functionalities of passive sensing data collection, such as tool performance, security, and privacy concerns, which could be crucial to data collection and application development processes. Therefore, future reviews should investigate how these aspects play a part in the use of passive sensing applications or wearables for depressive symptom screening in the elderly.

## Conclusion

This review found that the majority of these studies was conducted in western countries and applied cohort study designs. The most popular passive sensing data were related to sleep and physical activity measured via actigraphy. Sleep characteristics, such as wake after sleep onset time, and low level of physical activity, are significantly related to depression. Overall limitations on the generalizability, validity and reliability of measurements have to be improved, including follow-up periods, data measurements and confounding factors in cohort studies. Passive sensing data such as sleep, and physical activity parameters should be promoted as a tool for depression detection. However, the tool performance, security and privacy concerns should be explored in further research. Also, studies that exercise methods to ensure causal relationship between valid sleep and physical activity parameters with depression is of huge merit to expand the knowledge in this field and enhance its academic richness.

## Supporting information

S1 Checklist (DOCX)

S1 Table Summary of reviewed studies’ characteristics. (DOCX)

S2 Table Summary of reviewed studies’ data collection. (DOCX)

S3 Table Summary of reviewed studies’ data measurement and data analysis, results, limitations, and recommendations. (DOCX)

S4 Table Summary of reviewed studies’ quality assessment. (DOCX)
