# Supplementary material for: Depressive symptom screening in elderly by passive sensing data of smartphones or smartwatches: A systematic review
Source: PLoS One. 2024 Jun 27;19(6):e0304845. doi: 10.1371/journal.pone.0304845 (PMC11210876; doi:10.1371/journal.pone.0304845)
Supplement: S1 Table — (DOCX) [file pone.0304845.s002.docx]

**S1 Table** Summary of reviewed studies’ characteristics

| **Author** | **Paper name** | **Year** | **Country** | **Setting** | **Study design** | **Study period** | **Objective(s)** |
| --- | --- | --- | --- | --- | --- | --- | --- |
| Choi et al. (2022) | Depressed mood prediction of elderly people with a wearable band | 2022 | South Korea | Social welfare center | Cohort | 71 days | (1) To extract features from the E4 Wearable band related to the depression symptoms based on Diagnostic and Statistical Manual of Mental Disorders (DSM-V). Photoplethysmography (PPG), accelerometer sensor (ACC), electrodermal activity sensor (EDA), and temperature sensor (TEMP) data are related to depressed mood, fatigue or loss of energy, thoughts of death or suicidal ideation, and change in weight appetite symptoms (2) To use the proposed models to predict the general elderly people with depressed mood (3) To generate a general model and personal models |
| Cabanas-Sánchez et al. (2021) | Cross-sectional and prospective associations of sleep, sedentary andactive behaviors with mental health in older people: a compositional data analysis from the Seniors-ENRICA-2 study | 2021 | Madrid, Spain and four large adjacent cities (Getafe, Torrejón, Alcorcón and Alcalá de Henares) | Community | Cross-sectional | wave 0: 2015 and 2017 and wave 1: 2018 and 2019 | (1) To examine the cross-sectional associations of accelerometer-derived sleep, sedentary behavior (SB), light physical activity (LPA) and moderate-to-vigorous physical activity (MVPA) with mental health indicators (i.e., depression symptoms, loneliness, happiness and global mental health) in older people (2) To assess the prospective relationships between time-use composition and changes in mental health indicators in this same population |
| Vesel et al. (2020) | Effects of mood and aging on keystroke dynamics metadata and their diurnalpatterns in a large open-science sample: A BiAffect iOS study | 2020 | The US | Community | Cohort | 15 months | (1) To investigate the effects of mood, age, and diurnal patterns on naturalistic real-world typing (keyboard typing) acquired using a mobile health (mHealth) smartphone technology in a citizen science sample |
| Hoyos et al. (2020) | Circadian rhythm and sleep alterations in older people with lifetime depression: a case-control study | 2020 | Sydney, Australia | Clinical center/Health center | Case-control | 2 weeks | (1) To examine circadian rhythms in older patients with depression, with the primary outcomes of interest being dim light melatonin onset and phase angle of entrainment (2) To measure sleep architecture and quality assessed both objectively and subjectively (3) To examine associations between clinical correlates and circadian rhythmicity in an exploratory analysis to understand possible future clinical and therapeutic targets of major depressive disorder |
| Kim et al. (2019) | Depression Prediction by Using Ecological Momentary Assessment, Actiwatch Data, and Machine Learning: Observational Study on Older Adults Living Alone | 2019 | South Korea | Clinical center/  Health center | Cohort | 2 weeks (May 2017 to January 2018) | To develop a machine learning algorithm to predict the classification of depression groups among older adults living alone focusing on diverse data collected through a survey, an Actiwatch, and an Ecological Momentary Assessment (EMA) report related to depression |
| Aubourg et al. (2019) | Association between social asymmetry and depression in older adults: a phone call detail records analysis | 2019 | France | Community | Cohort | 12 months | (1) To investigate the existence of a general phone call habit in the older adult regarding the direction of his phone calls (2) To investigate the existence of a relation between such a habit and the depressive health state of the individual |
| Asai et al. (2018) | Farming habit, light exposure, physical activity, and depressive symptoms. A cross-sectional study of the HEIJO-KYO cohort | 2018 | Nara, Kansai Region, Japan | Community | Cross-sectional | September 2010 to March 2014 | (1) To objectively measured daytime light exposure and physical activity in real life situations with elderly participants (2) To investigate the association between farming habits and the prevalence of depressive symptoms |
| O'Brien et al. (2016) | A study of wrist-worn activity measurement as a potential real-world biomarker for late-life depression | 2016 | Northeast of England | Clinical center/  Health center | Cohort | 7 days | To explore the utility of a bespoke activity monitor to characterize activity profiles in Late Life Depression (LLD) more precisely |
| Alcántara et al. (2016) | Sleep disturbances and depression in the multi-ethnic study of atherosclerosis | 2016 | The US:( Baltimore City and Baltimore County, Maryland (MD); Chicago, Illinois (IL); Forsyth County, North Carolina (NC); Los Angeles County, California (CA); Northern Manhattan and the Bronx, New York (NY); and St. Paul, Minnesota (MN)) | Community | Cross-sectional | 7 days | (1) To examine the association of objectively and subjectively measured sleep disturbances (i.e., short sleep duration, symptoms of insomnia and excessive daytime sleepiness (EDS), sleep apnea syndrome) with depression among community-dwelling adults enrolled in the Multi-Ethnic Study of Atherosclerosis (MESA) Sleep Ancillary Study  (2) To investigate in exploratory analyzes whether race/ethnicity, socioeconomic status (education and income), and sex were significant effect modifiers |
| Luik et al. (2015) | 24-hour Activity rhythm and sleep disturbance in depression and anxiety: a population-based study of middle and older persons | 2015 | Ommoord district, Rotterdam, Netherlands | Community | Cross-sectional | December 2004 to April 2007 | (1) To investigate whether 24-hr activity rhythms and sleep are associated with depressive symptoms (2) To investigate whether 24-hr activity rhythm and sleep are associated with anxiety disorder (3) To investigate whether the 24-hr activity rhythm and sleep are each associated independently and specifically with depressive symptoms and anxiety disorders |
| Smagula et al. (2015a) | Circadian rest-activity rhythms predict future increases in depressive symptoms among community-dwelling older men | 2015 | The US (Birmingham, Alabama; the Monongahela Valley near Pittsburgh, Pennsylvania (PA); Minneapolis, MN; Palo Alto and San Diego, CA; and Portland, Oregon (OR)) | Clinical center/  Health center | Cohort | 16 months (December 2003 to March 2005) | To examine whether baseline Circadian rest-activity (CAR) disturbances are a risk factor for future increases in depressive symptoms |
| Maglione et al. (2014a) | Subjective and objective sleep disturbance and longitudinal risk of depression in a cohort of older women | 2014 | The US (Portland, OR; Minneapolis, MN; Pittsburgh and Monongahela Valley, PA; Baltimore, MD) | Clinical center/  Health center | Cohort | 12 months (February 1997 to February 1998) | To explore the longitudinal relationship between baseline subjectively and objectively assessed sleep measures and future depressive symptoms in a group of older women who reported few or no depressive symptoms at baseline |
| Maglione et al. (2012) | Depressive symptoms and subjective and objective sleep in community-dwelling older women | 2012 | The US (Portland, OR; Minneapolis, MN; Pittsburgh and Monongahela Valley, PA; Baltimore, MD) | Clinical center/  Health center | Cross-sectional | January 2002 and February 2004 | To examine the relationship between depressive symptoms and subjective and objective sleep in older women. We hypothesized that older women who endorsed more depressive symptoms would be more likely to report subjective sleep disturbances and more likely to have objective evidence of disturbed nighttime sleep and increased daytime napping |
| Palmius et al. (2017) | Detecting bipolar depression from geographic location data | 2017 | Oxford, the UK | Community | Cohort | 3 months (May to July 2015) | To detect clinically significant levels of depression using features extracted from geographic location recordings in a community Cohort of bipolar patients |
| Pye et al. (2021) | Irregular sleep-wake patterns in older adults with current or remitted depression | 2021 | Sydney, Australia | Clinical center/  Health center | Case-control | NA | (1) To assess sleep regularity in older adults with remitted and current major depression, relative to healthy controls (2) To examine rest-activity patterns using measures derived from non-parametric and cosinor analyzes (3) To explore the demographic, clinical, and medical correlates of altered sleep regularity and rest-activity patterns |
| Gruenenfelder-Steiger et al. (2017) | Physical activity and depressive mood in the daily life of older adults | 2017 | Neumünster, Switzerland | Community | Cohort | 7 days | (1) To focus on within-person associations between daily depressive mood and daily physical activity in older adults across 7 days (2) To explore the role of daily need-fulfillment associated with depressive mood  (3) To test the extent to which need-fulfillment explains the association between daily physical activity and depressive mood by means of a within-per-son mediation |
| Maglione et al. (2014b) | Depressive symptoms and circadian activity rhythm disturbances in community-dwelling older women | 2014 | The US (Portland, OR; Minneapolis, MN; Pittsburgh and Monongahela Valley, PA; Baltimore, MD) | Community | Cross-sectional | January 2002 and February 2004 | To examine the relationship between levels of depressive symptoms and disruption of circadian activity rhythms cross-sectionally in a large group of community-dwelling older women |
| Paudel et al. (2013) | Sleep disturbances and risk of depression in older men | 2013 | The US (Birmingham, Alabama; the Monongahela Valley near Pittsburgh, PA; Minneapolis, MN; Palo Alto, CA; San Diego, California; and Portland, OR) | Community | Cohort | 16 months (December 2003 to March 2005) | To test the hypothesis that objective sleep/wake disturbances, as well as self-reported sleep disturbances are associated with incident depression in older men |
| Abbas et al. (2022) | Utility of actimetry to detect apathy in old-age depression: a pilot study | 2022 | France | N/A | Cohort | 3 days | To evaluate the utility of actimetry (six features of daily activity measured by a wrist-worn accelerometer) to distinguish the activity of apathetic depressed elderly from non-apathetic depressed ones and control population |
| Lee et al. (2014) | Physical activity and depressive symptoms in older adults | 2014 | The US | Community | Cross-sectional | 2005 to 2006 | (1) To utilize objectively measured physical activity to determine more precisely the relationship between physical activity and depressive symptoms in older adults (age > 60 years) (2) To explore associations between selected demographic factors and physical activity |
| Smagula et al. (2015b) | Latent activity rhythm disturbance sub-groups and longitudinal change in depression symptoms among older men | 2015 | The US (Birmingham, AL; Minneapolis, MN; Palo Alto, CA; Monongahela Valley, PA; Portland, OR; and San Diego, CA) | Clinical center/Health center | Cohort | December 2003 to March 2005 | To further clarify the relationship between activity rhythm disturbances and the course of depression among older men, we tested whether any of the data-derived sub-groups experienced greater depressive symptom increases over 5 years. |

Note: Not applicable (N/A)
