# Supplementary material for: Depressive symptom screening in elderly by passive sensing data of smartphones or smartwatches: A systematic review
Source: PLoS One. 2024 Jun 27;19(6):e0304845. doi: 10.1371/journal.pone.0304845 (PMC11210876; doi:10.1371/journal.pone.0304845)
Supplement: S2 Table — (DOCX) [file pone.0304845.s003.docx]

**S2 Table** Summary of reviewed studies’ data collection

| **Author** | **Target group** | **Sample size** | **Sampling technique** | **Inclusion** | **Exclusion** | **Type of device** | **Name and function of application(s) used** | **Data collection** |
| --- | --- | --- | --- | --- | --- | --- | --- | --- |
| Choi et al. (2022) | Mean age: 76.0 | 14 | Convenience sampling | (1) Aged ≥ 65 years old (2) Must not have a history of depression diagnosis (3) Must live alone (4) Must be registered at a local social welfare center | N/A | Wearable | The Empatica E4 wearable band was used to measure heart rate, time domain features, frequency domain features, XYZ axis, moving/stationary states, skin activity statistic features, temperature statistic features. | Participants wore the E4 band for 67 hours a week on average. They may remove the band when they work with water or when they sleep or recharging the band. |
| Cabanas-Sánchez et al. (2021) | mean age (SD): 71.7 (4.3) | 1,679 | Sex- and district-stratified random sampling | (1) Community-dwelling individuals aged ≥ 65 years  (2) Must be in the city of Madrid and four large adjacent cities (Getafe, Torrejón, Alcorcón and Alcalá de Henares) | Died or loss follow up | Wearable | The actiGraph GT9X was used to assess time spent on sleep, sedentary behavior, light physical activity, and moderate to vigorous physical activity. | Participants wore the ActiGraph GT9X on the non-dominant wrist using a watch band for seven consecutive days. They were asked to only remove it during water-based activities. Days when the accelerometer registered at least 16 hours were considered as valid, and only results from participants with at least four valid days were included in the analyses. |
| Vesel et al. (2020) | Mean age (SD): 37.8 (12.3) (sample A) and 39.5 (11.9) (Sample B) | Sample A (n = 142,202 sessions, 250 users) and Sample B (n = 86,541 sessions, 147 users) | Convenience sampling | N/A | N/A | Smartphone | The BiAffect keyboard was used to record timestamps of keypress events and their type (i.e., character, punctuation, backspace, autocorrect, or autosuggestion). | Operationally, one typing session was initiated when the keyboard was activated, and terminated after eight seconds of inactivity, or at the time of keyboard deactivation. Session duration was defined as the length of a typing session in seconds. |
| Hoyos et al. (2020) | Mean age (SD): 63.9 (10.8) (case) and 66.7 (9.1) (control) | 64 (Depressed patients = 34 and healthy controls = 30) | Purposive sampling | (1) Aged ≥ 50 years old (2) Met Diagnostic and DSM-IV criteria for lifetime major depressive disorder (3) Had a depressive episode within the last 5 years (4) Had been clinically stable on medication | (1) Had other psychiatric illness including bipolar disorder, history of stroke, neurological disorder, head injury with loss of consciousness > 30-min, medical conditions known to affect cognition (i.e., cancer) and diagnosis of dementia  (2) Had Mini-Mental State Examination (MMSE) Score <24  (3) Had current shift-workers, trans meridian travel within the prior 60-days, use of medication that may affect sleep and/or melatonin secretion such as beta-blockers or lithium | Wearable | An actigraph (Actiwatch Spectrum, Philips Respironics, OR) was used to evaluate habitual sleep onset, total sleep time and sleep midpoint were used to assess the participants day-to-day sleep patterns (circadian rhythm). | Participants did sleep diaries for two weeks as well undertake overnight PSG and circadian assessments on three consecutive nights within the research facility. |
| Kim et al. (2019) | Age range: 65-94 | 47 | Convenience sampling | (1) Aged 65 to 94 years (2) Had at least mild levels of depressive symptoms (score < 5 on the Short Geriatric Depression Scale) (3) Understood Korean (4) Lived as a single household in the community | (1) Had significant cognitive impairment  (2) Had a high risk of suicide according to the Korean versions of the MMSE and Crisis Triage Ration Scale | Wearable | A wrist-worn Actiwatch (Actiwatch Spectrum PRO, Philips Respironics) was used to measure physical activity and ambient light exposure. | Participants wore the Actiwatch on the non-dominant wrist all the time and were instructed to take it off only when taking a bath or for a few minutes as needed. Furthermore, participants were instructed that long sleeves should not cover the light sensor in the Actiwatch. Data were collected continuously in 30-second epochs for 14 consecutive days. |
| Aubourg et al. (2019) | Median age (age range): 84 (71–91) | 19 | Convenience sampling | N/A | N/A | Smartphone | Network communication operator provided CRDS that were collected on participants' personal phone(s). | N/A |
| Asai et al. (2018) | ≥ 60 | 1,005 | N/A | Aged ≥ 60 years old | Had missing information about their farming habits or missing Geriatric Depression Scale (GDS) scores | Wearable | Wrist actigraphy (Actiwatch 2; Respironics Inc., Murrysville, PA, USA) was used to measure physical activity. | Participants worn the wrist actigraphy on the non-dominant arm, not to covering the device with their clothing by a rubber band to fold their sleeves. We regarded values <1 lux as artifacts due to covering the sensor by clothing and these values were not used in this analysis. |
| O'Brien et al. (2016) | > 60 | 59 (depressed patients = 29 and healthy control = 30) | Purposive sampling | (1) Aged over 60 years (2) Fulfilled DSM-IV criteria for current major depression, as assessed using the Mini-International Neuropsychiatric Interview (MINI) (3) Recruited from secondary care services covering four geographically based secondary catchment areas across the North East of England  (case are who fulfilled the DSM-IV criteria for current major depression and control are no self-reported history of depression or current depression as measured using the MINI) | (1) Had evidence of a severe or unstable physical illness  (2) Had known cognitive impairment or dementia  (3) MMSE score <24  (4) Acquired brain injury or stroke  (5) Had recent history or current evidence of substance abuse (i.e., alcohol, drugs), uncorrected visual or auditory sensory deficits; and history of electroconvulsive therapy (ECT) in the past 6 months (clinical sample) or any history of ECT (control sample) | Wearable | The wearable monitor was a stand-alone, wrist-mounted device that measured physical activity via three accelerometers: physical activity, jerk, and entropy. | N/A |
| Alcántara et al. (2016) | Mean age: 68.2 | 1,784 | Purposive sampling | Had no evidence of clinical cardiovascular disease at enrollment | (1) Had a history of the positive airway pressure machine use due to use of an oral appliance and due to oxygen use  (2) Resided too far from the study sites to participate  (3) Had poor quality polysomnography data (4) Had missing covariate data  (5) Had incomplete questionnaire | Wearable | The Actiwatch Spectrum wrist actigraph (Philips Respironics, Murrysville, PA, USA) was used to measure mean sleep duration and sleep continuity. | Participants wore the Actiwatch on their non-dominant wrist for seven consecutive days. Actigraphic data were scored during 30-second epochs as sleep or wake by Actiware-Sleep version 5.59 analysis software. |
| Luik et al. (2015) | Mean age: 67.5 (agreed participants) and 62.3 (disagreed participants) | 1,714 | Purposive sampling | (1) Were in population-based cohort study of middle-aged and elderly inhabitants of Rotterdam, the Netherlands (2) Agreed to participate in the actigraphy study | (1) Did not consist of 4 days and nights data recordings (2) Collected in a week of daylight-saving time  (3) Malfunctioned Actiwatch  (4) No information on depressive symptoms  (5) Refused to answer all depressive questions  (6) Missing data due to refusal or time constraints | Wearable | Actigraphy was used to access the 24-hr activity rhythm (Actiwatch model AW4, Cambridge Technology Ltd., Cambridge, UK). Activity rhythms included the interdaily stability, intradaily variability, and dominant rest phase onset. | Participants wore the actigraph continuously for seven consecutive days and nights, and to remove it only while bathing (measured in 30-s epochs). |
| Smagula et al. (2015a) | ≥ 65 | 2,696 | Purposive sampling | (1) had ≥3 of 24-hour periods of technically adequate actigraph data (2) Could walk without assistance and were without bilateral hip replacements (3) Included only participants without baseline depression (GDS scores ≤ 2) | (1) Had regularly used overnight nocturnal oxygen therapy, positive pressure or oral appliances for treatment of sleep apnea.  (2) The reason of non-participation was excluded: death (n = 349), terminated study participation (n = 39), declined sleep study (n = 1997), or Osteoporotic Fractures in Men Study (MrOS) Sleep Study recruitment goals had already been met (n = 324). | Wearable | The Octagonal Sleep Watch actigraph (SleepWatch-O; Ambulatory Monitoring, Inc, Ardsley, NY) was used to estimate rest/activity rhythms including, rhythm height parameters, rhythm timing measures, and rhythm robustness. | Participants wore actigraphs on the non-dominant wrist for a minimum of five consecutive 24-hour periods except when bathing or during water sports. |
| Maglione et al. (2014a) | Mean age: 82.5 | 952 | Purposive sampling | (1) Were women participating in Study of Osteoporotic Fractures (SOF), visits 8 and 9 (around 15 and 20 years after the original assessment)  (2) Participated in objective assessment of sleep by actigraphy at visit 8 with successfully data collection. (3) Completed GDS questionnaire. | Required assistance with ambulation or had undergone bilateral hip replacement | Wearable | Actigraph (SleepWatch-O) was used to evaluate sleep and wake patterns via an accelerometer. | Participants wore the acitgraph on their nondominant wrist for at least three consecutive 24-hour periods. Movements were recorded and summarized in 1-minute epochs. They also completed sleep diaries for the time period they wore the actigraph, including information about times they got into and out of bed and times their actigraph was removed. |
| Maglione et al. (2012) | Mean age: 83.6 | 2,995 | Purposive sampling | (1) Were women enrolled in the SOF (ongoing, multi-center, prospective, observational cohort study of primarily Caucasian) | Required assistance with ambulation or had undergone bilateral hip replacement | Wearable | Actigraphs (SleepWatch-O, Ambulatory Monitoring, inc., Ardsley, NY) was used to measure objective sleep parameters: total sleep time (TST), minutes sleep, sleep efficiency, sleep onset latency, time awake after sleep onset, number of long wake episodes, and number nap episodes. | Participants wore the actigraphs on their non-dominant wrist for at least three consecutives 24-hour periods. |
| Palmius et al. (2017) | Mean age (SD): 42.0 (14.0) (control), 46.0 (14.0) (Case QIDS Score < 11), and 41.0 (15.0) (Case QIDS Score ≥ 11) | 49 (bipolar depression patients = 29 and healthy control = 20) | Convenience sampling | Were healthy without symptoms of mental disorders, or bipolar disorder (BD) or BD patients | N/A | Smartphone | A custom open-source smartphone (Android-based Samsung GALAXY S III or S4) app was used to collect behavioral data from participants and self-reported clinically validated questionnaires to collect psychiatric state. | Geographic location was collected using a custom app installed that records the anonymized geographic location of the phone without stated specific time frame. Participants were asked to complete the self-report Quick Inventory of Depressive Symptomatology questionnaire (QIDS-SR 16) on a weekly basis through the True Colors monitoring system. The questionnaire could be completed at any time before the next weekly prompt. |
| Pye et al. (2021) | Mean age (SD): 63.8 (8.6) | 138 (current major depression = 27, remitted major depression = 64 and healthy controls = 47) | Convenience sampling | (1) Had referral criteria to the Healthy Brain Ageing clinic require participants to have new onset subjective cognitive complaints and/or mood disturbance and a referral from a primary care or specialist physician (2) Included participants with baseline actigraphy assessment  (Case 1 were participants with a previous DSM-IV-defined diagnosis of Major Depressive Disorder (MDD) episode that had remitted (DEP-R); Case 2 were those who currently met DSM-IV criteria for a major depressive episode (DEP-C); Control are healthy elderly who never experienced a DSM-IV major depressive episode and did not meet the criteria for Mild Cognitive Impairment (MCI). | (1) Had been diagnosis of a neurological disease (i.e., epilepsy, Parkinson’s disease), diagnosis of bipolar disorder or schizophrenia, current or past history of substance abuse and dependence, and history of stroke (2) Had previous head injury with a loss of consciousness >30 minutes (3) Had inadequate English language skills for assessment | Wearable | Actigraph (Respironics Actiwatch Spectrum and Spectrum Pro devices) was used to measure rest-activity patterns (rest and wake periods, TST (min), wake after sleep onset (WASO; min), and the time of sleep onset and offset). | Participants wore the Actiwatch on their non-dominant wrist for the next 14 consecutive days, and to keep a daily sleep diary. Activity counts were collected in 30-second epochs, using Respironics Actiwatch Spectrum and Spectrum Pro devices. Small portions of missing data from watch removal due to bathing or other routine activities. |
| Gruenenfelder-Steiger et al. (2017) | Mean age (SD): 78.0 (7.9); Age range: 65-93 | 68 | Convenience sampling | (1) Were older adults with mobile and cognitively able to pass the MMSE (2) Did not need help with their daily activities from an outpatient service for homecare | N/A | Wearable | An accelerometer (movisens3; movisens GmbH, 2016) was used to measure physical activity objectively. This motion sensor records raw data on three-axes: acceleration, barometric altitude, and temperature. The accelerometer was also used to calculate the daily number of steps as the key parameter for daily physical activity. | Participants wore the accelerometer over a period of seven consecutive days. |
| Maglione et al. (2014b) | Mean age: 82.5 | 3,020 | Purposive sampling | (1) Were women participating in SOF, visits 8 and 9 (approximately 15 and 20 years after the original assessment)  (2) Participated in objective assessment of sleep by actigraphy at visit 8 with successfully data collection  (3) Completed GDS questionnaire | Required assistance with ambulation or had undergone bilateral hip replacement | Wearable | The actigraphy (SleepWatch-O, Ambulatory Monitoring, Inc., Ardsley, NY) was used to measure circadian activity rhythm computed from activity data. Circadian activity rhythm variables included acrophase (time of day of maximum modeled activity), amplitude (difference between the maximum and minimum of the modeled activity curve), mesor (mean of the modeled activity curve), left half-deflection (time of day women's activity pattern switched from low, below the mesor, to high, above the mesor), right half-deflection (time of day women's activity pattern switched from high to low), and pseudo-F-statistic. | Participants wore actigraphs on their nondominant wrist for at least three consecutive 24-hour periods. |
| Paudel et al. (2013) | Mean age (SD): 75.9 (5.3) | 2,510 | Purposive sampling | Were categorized as nondepressed at baseline (GDS score < 6) | Excluded men with a history of bilateral hip replacement and men who were unable to walk without the assistance of another person | Wearable | An octagonal sleep watch actigraph (Ambulatory Monitoring, Inc., Ardsley, NY) was used to measure sleep/wake parameters; sleep efficiency, sleep latency, and TST. | Participants wore the actigraph for a minimum of five nights, fastened around their nondominant wrist, to be removed only when bathing or during water sports. However, most participants did not complete the requested five nights of actigraphy data, the researcher restricted our primary analyses to men who had at least 72 hours of actigraphy recordings. |
| Abbas et al. (2022) | Age range: 67-87 | 37 | N/A | (1) Cases were clinically depressed participants, separating into non-apathetic and apathetic (2) Control were non-depressed participants | N/A | Wearable | The ActiGraph wGT3X-BT (Bluetooth Smart) was used to measure triaxial accelerometer and digital filtering technology. | Participants wore the actigraph over a period of 3 ±0.23 days during their daily routine. |
| Lee et al. (2014) | Mean age (95% CI): 70.4 (69.8 - 71.0) | 805 | Stratified multistage probability random sampling | (1) Aged ≥ 60 (2) Completed both objective physical activity measurement with an accelerometer and depression screening | N/A | Wearable | The accelerometer Actigraph AM-7164 (manufactured by ActiGraph of Ft. Walton Beach, Florida, USA) was used to measure physical activity. | Participants wore the actigraph for seven consecutive days with at least one valid day of monitoring in which the accelerometer was worn for at least 10 hours. |
| Smagula et al. (2015b) | Mean age (SD): 76.3 (5.5) | 2,933 | Purposive sampling | (1) Had ≥3 of 24 hour periods of technically adequate actigraph data (2) Could walk without assistance and were without bilateral hip replacements (3) Were required to have complete outcome data from baseline and at least one other time-point | (1) Had regularly used overnight nocturnal oxygen therapy, positive pressure or oral appliances for treatment of sleep apnea.  (2) The reason of non-participation were excluded: death (n = 349), terminated study participation (n = 39), declined Sleep Study (n = 1997), or because MrOS Sleep Study recruitment goals had already been met (n= 324). | Wearable | The Octagonal Sleep Watch actigraph (SleepWatch-O; Ambulatory Monitoring, Inc, Ardsley, NY) was used to estimate rest/activity rhythms including, rhythm height parameters, rhythm timing measures, and rhythm robustness. | Participants wore actigraphs on the non-dominant wrist for a minimum of five consecutive 24-hour periods except when bathing or during water sports. |

Note: Standard Deviation (SD); Confidence Interval (CI)
