# Supplementary material for: Depressive symptom screening in elderly by passive sensing data of smartphones or smartwatches: A systematic review
Source: PLoS One. 2024 Jun 27;19(6):e0304845. doi: 10.1371/journal.pone.0304845 (PMC11210876; doi:10.1371/journal.pone.0304845)
Supplement: S3 Table — (DOCX) [file pone.0304845.s004.docx]

**S3 Table** Summary of reviewed studies’ data measurement and data analysis, results, limitations and recommendations

| **Author** | **IV and measurement** | **DV and measurement** | **CV and measurement** | **Data analysis** | **Results** | **Limitations** | **Recommendations** |
| --- | --- | --- | --- | --- | --- | --- | --- |
| Choi et al. (2022) | (1) Heart rate, time domain features, frequency domain features were measured by the PPG sensor (2) X, Y, Z axis statistic features and moving, stationary states were measured by the ACC sensor (3) Skin activity statistic features were measured by the EDA sensor (4) Temperature statistic features were measured by the TEMP sensor, expressed in degrees on the Celsius | Depressive symptoms were measured by a Korean version of Patient Health Questionnaire (PHQ-9) and the Korean Version of Short Geriatric Depression Scale questionnaire (SGDS-K). The PHQ-9 was conducted once a week throughout the experiment. The SGDS-K is performed once at the beginning of the experiment to determine existing degree of depression. | N/A | The models we proposed predict whether the participant is in a normal state or a mild depression. A general model is developed to use as an objective indicator. We focused to adopt a machine learning model instead of deep learning. Eight classification algorithms are used for the model generation: K-NN classifier, Support Vector Machine, Decision Tree, Random Forest, Gradient Boosting, XGBoost, 3- Multi-layer Perceptron (MLP), 4-MLP. For personal modeling, models of ACC sensor, PPG sensor, and both sensors together were conducted. A random forest model was applied to each model. The train data of the ACC sensor consists of 154 statistical features and two motion features. The train data of the PPG sensor consists of frequency domain features related to emotion. ACC and PPG Model is generated with all the features from the two sensors. | The general model developed with 4-MLP has shown 69% accuracy and 78% recall and the k-NN model has shown 80% recall. The personal model is generated in three different modes by sensor data which are the ACC-Only Model, the PPG-Only Model and the ACC and PPG Model. The ACC-Only Model has shown the highest recall rate 82.7% in average. | (1) It has limitation of collecting certain data for the depressed participants. The participants have not been diagnosed with depression and have regular social activities. They might be in a good mood throughout the duration of the experiments. (2) The survey may not be trusted depending on the individual’s subjectivity and the situations at the time of the survey. This affected the PHQ-9 survey results to be more biased toward normal data while we tried to recognize the “mild depression” label as the depressed status. | N/A |
| Cabanas-Sánchez et al. (2021) | 24-hour time use composition (accelerometer-derived sleep, SB, LPA and MVPA were measured by the actiGraph GT9X . | Mental health outcomes:  (1) Depression was measured by the 10-item version of the GDS. (2) Loneliness was assessed with the three-item loneliness scale.  (3) Level of happiness was measured by the Cantril Ladder of Life Scale.  (4) Global mental health was measured using the Spanish version of the 12-item Short Form Health Survey (SF12, version 2). | Self-administered questionnaire:  (1) Demographics (sex, age, educational level, marital status , household economy)  (2) Tobacco smoking (3) Alcohol consumption  (4) Height and weight, body mass index (BMI)  (5) Diseases diagnosed by a physician (6) Food consumption and total energy intake were collected by a validated computer-assisted face-to-face diet history. (7) Cognitive function was measured by the MMSE usual gait speed (the 8-feet test, following the standardized protocol from the Short Physical Performance Battery). | We fitted regression models using time-use composition as explanatory variables and the mental health indicators at wave 0 as response variables. Similarly, for prospective analyses, the set of ilr coordinates of the time-use composition was used as explanatory variables, while the change in mental health indicators was considered as dependent variables in regression models. Tree models were created with progressive adjustment for potential confounders. In prospective analyses, regression models were additionally adjusting for baseline values of each mental health indicator. | (1) In cross-sectional analyses at wave 0 (n = 2489), time-use composition as a whole was associated with depression and happiness (all p < 0.01). The time spent in MVPA relative to other behaviors was beneficially associated with depression (γ = -0.397; p<0.001), loneliness (γ = -0.124; p = 0.017) and happiness (γ = 0.243; p < 0.001). (2) During the follow-up in the fully adjusted models, the time spent in MVPA relative to other behaviors was associated with favorable changes in global mental health (γ = 0.892; p = 0.049), while sleep time relative to the rest of behaviors was related to adverse changes in depression (γ = 0.347; p = 0.049). | (1) It has a relatively short follow-up period, and the observational nature of the study, which does not allow for entirely discarding residual confounding. (2) It uses validated tests to assess a wide range of mental health constructs, but the diagnoses by a physician were not available. (3) It does not permit to establish the optimal doses of each time-use behavior to maintain or improve mental health in older people. | N/A |
| Vesel et al. (2020) | Mood ratings was measured by PHQ-8. | Keyboard typing features was measured by an mHealth application dependent variables of session-level typing speed, typing variability, typing accuracy, and session duration and their relationship to other session-level features and demographics. | Self-administered questionnaire:  (1) Demographics (age and gender)  (2) Psychiatric disorders | We conducted growth curve mixed-effects (multilevel) models in R and lme, using maximum likelihood fitting to examine dependent variables. The intraclass correlation coefficient (ICC) measures the homogeneity of the cluster on the dependent variable and is calculated for our 2-level model intercepts only as the ratio of between cluster variance to total variance for the random effects model. The fixed-effects structure was tested in 2 ways. (1) Fixed effects were constructed hierarchically through the addition of increasingly complex terms, and likelihood ratio tests were used to compare nested models. (2) The significance of individual predictors was assessed via estimating the degrees of freedom with Satterthwaite approximations. | (1) More severe depression relates to more variable typing speed (p < 0.001), shorter session duration (p < 0.001), and lower accuracy (p < 0.05). Moreover, typing speed and variability exhibit a diurnal pattern, being fastest and least variable at midday. Older users exhibit slower and more variable typing, as well as more pronounced slowing in the evening. (2) The effects of aging and time of day did not impact the relationship of mood to typing variables and were recapitulated in the 250-user group. | (1) Mood self-reports were sparser than keyboard data, so we elected to propagate their scores to typing sessions within a time window to preserve as much typing data as possible.  (2) Many enrolled participants did not contribute enough keypresses to be included in the analysis. (3) Cognition spans multiple recognized domains, yet it remains to be seen how our passive measurements may potentially map onto these distinct domains. Moreover, data collection in the wild is more susceptible to inattentiveness and distractions, potentially diluting the predictive strength of our metrics. | N/A |
| Hoyos et al. (2020) | Depression was diagnosed by a structured clinical assessment by an Old Age Psychiatrist. Lifetime and current depression were confirmed using the affective component of the Structured Clinical Interview for DSM-IV-R. The Hamilton Depression Rating Scale was used to determine depression severity | (1) Objective sleep was measured by PSG and actigraphy. (2) Circadian assessment was measured by actigraphy. (3) Subjective quality of sleep was measured by the PSQI to assess sleep quality over the previous month. | Self-administered questionnaire:  (1) Demographics (age, gender, years of education) (2) Global cognition was collected by MMSE Score. (3) BMI or level of medical illness burden were collected by Cumulative Illness Rating Scale. | All data are reported as means and standard deviations unless otherwise stated. Between group comparisons were performed using independent samples t-tests, Mann-Whitney U tests or Chi-squared tests where warranted. Normality of outcome variables was determined by visual inspection of histograms and then Pearson and Spearman correlation coefficients were used as appropriate. Due to the small amount of missing data, data imputation methods were not utilised. All analyses were two-tailed and employed an alpha level of 0.05. | (1) Older people with largely remitted lifetime depression have a longer phase angle of entrainment relative to healthy controls. (2) Subjective and aspects of objectively assessed sleep differed in those with lifetime depression. (3) Found that those with depression had longer Rapid Eye Movement (REM) latency and longer sleep latency than healthy control participants (p < 0.05). | (1) It cannot draw causal inferences. (2) It may not be generalizable to all older people with lifetime depression since we studied a specific population with generally remitted depression symptoms and additionally had to exclude many other populations due to the nature of the outcomes. | (1) It should explore if earlier dim light melatonin onset is clinically relevant in this population. (2) It should explore optimal maintenance treatments for those with remitted symptoms and persistent sleep-wake disruption is now needed. |
| Kim et al. (2019) | Physical activity and ambient light exposure were measured using a wrist-worn Actiwatch. | Depression was measured by the SGDS-K and the Korean version of the Hamilton Depression Rating Scale (K-HDRS) | N/A | Compared both groups’ 4-hour mean differences in the selected variables using a Mann-Whitney U test and time series plot. To find additional variables to consider for the prediction model, we tested diverse types of sleep parameters, such as total time in bed, TST, sleep efficiency, and WASO. We selected EMA score, activity, and ambient light exposure and sleep efficiency to perform a binary logistic regression. Data were divided into training data and test data, and the machine learning method was applied to calculate the predictive power of the model. Data were split at a 0.65:0.35 ratio for training and testing, respectively. Each indicator was calculated in the Confusion matrix. In a participants' activity timeseries, continuous data were collected by the Actiwatch every 30 seconds. Triaxial data were calculated as an activity count. On the basis of the characteristics of signal data collected in the time series at 30-second intervals, null or abnormal values because of user or device error were checked and excluded from the analysis. | On the basis of the SGDS-K and K-HDRS, 38% (18/47) of the participants were classified into the probable depression group. They reported significantly lower scores of normal mood and physical activity and higher levels of white and red, green, and blue (RGB) light exposures at different degrees of various 4-hour time frames (all p < 0.05). Sleep efficiency was chosen for modeling through feature selection. Comparing diverse combinations of the selected variables, daily mean EMA score, daily mean activity level, white and RGB light at 4:00 pm to 8:00 pm exposure, and daily sleep efficiency were selected for modeling. Conventional classification based on binary logistic regression had a good model fit (accuracy: 0.705; precision: 0.770; specificity: 0.859; and area under receiver operating characteristic curve (AUC): 0.754). Among the 4 machine learning models, the logit model had the best fit compared with the others (accuracy: 0.910; precision: 0.929; specificity: 0.940; and AUC: 0.960). | (1) Two screening instruments were used that relied on subjective reports to classify individuals in the two depression groups because these instruments had been widely used in community settings. However, a medical diagnosis based on DSM-V may be needed to define the clinically diagnosed depression group for data processing and representation. (2) Generalization of the EMA data and levels of activity is limited because every individual has different levels of normal with reference to a depressed mood or inactivity (3) The limited generalizability is expected for depressed men in older populations because the majority of older adults were women in Korea. Thus, it is necessary to oversample the groups of men for the next study on this topic. | N/A |
| Aubourg et al. (2019) | Phone call detail records (CDRs) were collected on their personal phone(s) and provided by their network communication operator. Each phone call detail record contains the date, hour, source used-ID, destination user-ID, direction and duration of the call (in seconds). Additionally, individuals having several phones registered by their network communication operator, for instance one or more landline phone(s)and/or one or more mobile phone(s), provided CDRs for all of them | Depression was measured by GDS by a health professional every 3 month. | Demographics (sex and age) | CDRs data are presented in the form of monthly First, the evaluation of balance in the older individuals phone call activity on his outgoing and incoming phone calls then distinguish users into 3 phone behaviors. Compare the differences between the monthly number of outgoing phone calls and the monthly number of incoming ones, for each individual, using Wilcoxon signed-rank tests. The association between the outgoing phone call activity and the incoming phone call activity was analyzed Pearson's correlation coefficient. Bland-Altman plots are then used to evaluate the agreement between both of these phone call activities. Verify three different distributions using the Kruskal-Wallis comparisons test for each of these asymmetry indicators. Second, measure the relation between both of the asymmetry indicators and the older individuals GDS score by means of the Pearson's correlation coefficient. The level of significance was set as p<0.05. | We found significant correlations between asymmetry and the depressive state assessed in the older individual. Particularly, (1) reactive users are more depressed than the others, and (2) not depressed older adults tend to be proactive (p < 0.05). | It may not be able to generalize to the overall population since the sample is small (n = 26) | It should investigate the phone call direction parameter by combining CDRs analysis with health data in older adults, whether and how similar results could be observed under different conditions, or in different sets of data. This could imply working on larger datasets, but also, more broadly, in leading analyses on different populations, such as young, disabled, or chronically ill individuals for instance. |
| Asai et al. (2018) | Physical activity and daytime light exposure were measured by wrist actigraphy (Actiwatch 2; Respironics Inc., Murrysville, PA, USA) | Depression was measured by the GDS score ≥ 6. | Self-administered questionnaire: (1) Demographics (age, gender, education length, house-hold income, living alone) (2) Current smoking and Ethanol intake (3) BMI (4) Disease (Hypertension/Diabetes) (5) Daytime systolic BP measured using a validated device  (6) Bedtime and rising time (7) Farming habits | The means of continuous variables with normal distributions and proportions compared using t-test and chi-square tests, respectively. Comparisons of variables with normal and skewed distributions, among the three groups, were conducted using the Dunnett and Steel tests, respectively. Crude and adjusted odds ratios (OR and AOR) for depressive symptoms were estimated using a logistic regression model. | Farming habit with long duration (>7.0 h/week) showed significantly lower OR for depressive symptoms (AOR = 0.63, 95% CI = 0.41-0.96) compared with participants without farming habit independent of confounders such as age, gender, body mass index smoking, drinking, daytime ambulatory systolic blood pressure, diabetes, living alone, education, income, and daylength. Even in farming with short duration (≤7.0 h/week), we found significant association with lower OR for depressive symptoms (AOR = 0.64, 95% CI = 0.42-0.97). Light exposure and daytime physical activity measured by wrist actigraphy were significantly higher among participants with longer farming habits (p for trend <0.01). Physical activity mediated 12.0% of association between farming habit and depressive symptoms. | A cross-sectional association may be found because the participants with depressive symptoms tended to avoid farming. A longitudinal study is warranted to determine the direction of causality. | The association was significant even in farming with short duration, and it may potentially suggest an easy preventive measure for depression. |
| O'Brien et al. (2016) | Physical activity was measured by the stand-alone, wrist-mounted device wearable monitor. | Depression was measured by the GDS-15. | Self-administered questionnaire: (1) Demographics (age) (2) BMI (3) Pre-morbid IQ were removed from the estimated measures through a linear regression of each variable with confounding variables | Group differences were assessed using independent t-tests. To militate against the problem of multiple comparisons with the neuropsychological data, composite average Z scores were created for five conceptual domains. Potentially confounding effects of age and BMI and pre-morbid IQ were removed from the estimated measures through a linear regression of each variable with confounding variables. | Physical activity was significantly reduced in LLD compared with controls (t = 3.63, p < 0.001), primarily in the morning. LLD subjects showed slower fine motor movements (t = 3.49, p < 0.001). In LLD patients, activity reductions were related to reduced ADL (r = 0.61, p < 0.001), lower Quality of Life (QoL) (r = 0.65, p < 0.001), associative learning (r = 0.40, p = 0.036), and higher Montgomery–Åsberg Depression Rating Scale score (r = −0.37, p < 0.05) | It does not able to obtain MR imaging (MRI) scans for all subjects. The study was cross-sectional; hence we cannot determine causality in the relationship between physical activity and the other key variables included in our comparisons. It is important to bear in mind that some elements of the relationship between physical functioning, depression and cognitive deficits may be bi-directional and interrelated. | (1) Higher resolution analysis of accelerometer-derived physical activity may provide a suitable surrogate marker for depression in older adults.  (2) Further evidence is needed to assess the long-term adherence for bespoke devices.  (3) Other developing technologies may be more appropriate for investigating the link between these variables in LLD. |
| Alcántara et al. (2016) | (1) Objective sleep: sleep duration and sleep continuity was measured by Spectrum wrist actigraph. (2) Self-reported sleep diary information and data on light from the wrist actigraphy were used to annotate the records, and determine a lights-off period and sleep onset latency. (3) PSG was used to measure sleep architecture and Obstructive Sleep Apnea, it provided quantitative assessments of levels of overnight hypoxemia, apneas and hypopneas, and sleep stage distributions.  (4) Subjective sleep: insomnia was measured using the Women Health Initiative Insomnia Rating Scale (WHIIRS).  (5) Sleepiness was measured using the Epworth Sleepiness Scale (ESS). Sleep apnea syndrome was defined as apnea-hypopnea index (AHI) > 15 plus ESS > 10. | Depressive symptoms at Exam 5 were measured by the Center for Epidemiologic Studies-Depression (CES-D). | Self-administered questionnaire: (1) Demographics (sex and race/ethnicity, education, and total gross family income) (2) Current smoker status  (3) Frequency of alcohol consumption at Exam 5  (4) BMI was calculated from measured weight and height.  (5) Hypertension at Exam 5.  (6) Diabetes or self-reported use of a diabetes medication. (7) Medication use was assessed in-home using a standard medication inventory approach.  (8) Cardiovascular disease from medical records by study physicians. | Computed means or proportions of descriptive characteristics for the overall sample and by depression status. For the primary analyses, we used relative risk regression (a generalized linear model with log link, Gaussian error structure, and robust standard error estimates) to compute the prevalence ratio (PR) of depression associated with each sleep measure. Prior to fitting models, we used to generalize additive models to test potential nonlinearity of the association of sleep measures with depression. To determine the robustness of our findings, we conducted five sensitivity analyses. | (1) The higher depression PR associated with long sleep duration were attenuated after adjustment for the full set of covariates, but the estimate associated with short sleep duration remained statistically significant (PR = 1.47; 95% CI = -1.94). With regard to measures of sleep continuity, PSG assessed low sleep maintenance efficiency was associated with 47% increase in the PRs of depression in site- and age-adjusted models (PR = 1.47; 95% CI = 1.08-2.01). (2) Those who spent less than 10% in REM sleep had increased prevalence of depression (PR = 1.69; 95% CI = 1.23-2.33) when compared to those with an intermediate proportion of REM sleep duration (10–24.9%) in site- and age-adjusted models. The adverse association of low proportion of REM sleep with depression remained significant in models that further adjusted for demographics, education, and income (PR = 1.71; 95% CI = 1.22-2.41), as well as behavioral risk factors and medical conditions (PR = 1.57; 95% CI = 1.08-2.27). Similarly, those with a high proportion of REM sleep (25% or more) had an increased prevalence of depression (PR = 1.42; 95% CI = 1.03-1.95) relative to those with average proportion of REM sleep in fully adjusted models. Excessive daytime sleepiness (EDS) had a 62% increased prevalence of depression (PR = 1.62; 95% CI = 1.23-2.13) relative to those without EDS.  (3) Among those with more than a high school education, short sleep duration relative to average sleep duration was associated with a higher prevalence of depression (PR = 1.61; 95% CI = 1.09-2.36). Sex-stratified models indicated that among men, insomnia was associated with a markedly high PR for depression (PR = 2.51; 95% CI: 1.68, 3.75). | (1) It is not able to assess causality and directionality. (2) Although we used a well-validated self-report scale to assess depressive symptoms, it is possible that depression classifications might differ with use of a clinical psychiatric interview. (3) Though we had specific hypotheses about the association of sleep disturbances and depression and potential sociodemographic effect modifiers, this resulted in multiple comparisons. (4) PSG was conducted over 1 night and thus may not be reflective of typical sleep and further subject to “first-night” effects. | (1) We should use prospective cohort designs to determine whether sleep disturbances are early prodromal or prognostic indicators of depression and more confidently establish temporality. Additionally, further understanding the psychobiological mechanisms linking these different dimensions of sleep to depression, or depression to sleep, are important to explore.  (2) We should replicate these findings using larger multiethnic samples that are adequately powered to detect significant interactions and with multimethod assessments of depression as well as related psychosocial factors known to affect sleep and depression (i.e., discrimination, occupational stress) |
| Luik et al. (2015) | (1) Objective sleep/wake parameters were measured by Actiwatch model AW4. The duration participants wore the actigraph (time into, time out, time when the actigraph was removed) and was measured by sleep diaries. (2) Subjective sleep quality and sleep disturbances over the past month were measured by the PSQI and the ESS. | (1) Depression was assessed by a home-interview using the CES-D scale. Participants who screened positive for depressive symptoms underwent a semi structured psychiatric interview with the Schedules for Clinical Assessment in Neuropsychiatry. (2) Anxiety disorders was assessed by the Munich version of Composite International Diagnostic Interview (M-CIDI). | Self-administered questionnaire: (1) Demographics (sex, age, partnership, employment status, and education)  (2) Alcohol use, coffee use, and current smoking  (3) Sleep characteristics: duration of actigraphy and possible apnea  (4) medical status:  (4.1) Cognitive status was measured by the MMSE (4.2) Activities of daily living (ADL) was measured by the Stanford Health Assessment Questionnaire (4.3) BMI during a center visit  (5) Use of psycholeptics, use of psychoanaleptics, use of sleep medication during actigraphy, and use of medication prescribed for blood and blood-forming organs, cardiovascular system, genito-urinary system and sex-hormones, systemic hormonal preparations, and the respiratory system were measured by interview | 24-hr activity rhythm and sleep were associated with depressive symptoms and anxiety disorders  (1) adjusted for sex and age (2) adjusted for partnership, employment status, cognitive status, ADL, psycholeptics, psychoanaleptics, sleep medication, BMI, and coffee use. We only included the a priori selected covariate in the model if the covariate predicted depressive symptoms or anxiety (p < .10) (3) assess whether activity rhythms and sleep were independent predictors of depressive symptoms and anxiety, we studied the associations in a mutually adjusted model. The variance inflation factor (VIF) indicated that there was no multicollinearity (VIF < 2). | (1) Actigraphically assessed disturbances of the circadian rhythm and sleep were related to depressive symptoms and anxiety disorders. The stability, fragmentation, and timing of the activity rhythm were all related to depressive symptoms. Of the sleep characteristics, only WASO and self-rated sleep quality were related to more depressive symptoms.  (2) Circadian rhythm disturbances and sleep were related to depressive symptoms with anxiety disorder | (1) It cannot draw any conclusions on temporality since our study is cross- sectional. (2) It lacks the precision of polysomnography since the observed circadian rhythms is an indirect assessment only.  (3) There is a limited statistical power of analyses because of the small number of cases with MDD (n = 22) (4) There is a combination of different anxiety disorders following the DSM-IV-TR, however our results suggest that the mechanisms underlying the individual anxiety disorders are not the same. | N/A |
| Smagula et al. (2015a) | Sleep-wake patterns were measured by the Octagonal Sleep Watch actigraph from movement using a piezoelectric biomorph-ceramic cantilevered beam, which generates a voltage each time the actigraph is moved. | Depressive symptoms at base line and follow up were measured by the GDS-15. | - Self-administered questionnaire: (1) Demographics (educational achievement) (2) The extent to which they received a physician diagnosis of the medical conditions  (3) Smoking status, caffeine, and alcohol use (4) BMI  (5) Mental health: cognitive function was measured using the 3MS, Anxiety symptoms were measured using the validated Goldberg Anxiety Scale.  (6) Sleep: The PSQI was used to measure daytime sleepiness. Participants also completed the ESS. Sleep disordered breathing (SDB) was measured using one night of in-home PSG. (7) Physical activity was measured by The Physical Activity Scale for the Elderly (PASE).  (8) Activity of Daily Living impairment was measured by IADL.  (9) Participants were asked to bring all medications used within the last 30 days. | Univariate comparisons of covariates across levels of baseline depressive symptoms categories were made using ANOVA or Kruskal Wallis tests for continuous variables and chi-squared homogeneity tests for categorical variables. CAR parameters may have non-linear relationships with depression. | Community-dwelling older men with lower amplitude, later activity peaks, and less robust CARs were more likely to have prevalent clinically significant depressive symptoms. We also found that men with less robust rhythms had higher odds of experiencing symptom increases from minimal to both mild and clinically significant levels. This association was independent of multiple confounders including chronic diseases, self-reported physical activity, sleep disturbances, and other relevant covariates. | (1) It cannot be generalizable to other populations since our study’s sample consisted of only older men free from cognitive impairment  (2) It has a relatively short follow-up period excludes assessment of the durability of this risk, and these effects may be observed only acutely.  (3) There are unmeasured confounders. (4) The CAR is not a direct indicator of circadian biology although it reflects the activity of the master biological keeper in the suprachiasmatic nucleus. The CAR can be readily “masked” by voluntary behavior. | N/A |
| Maglione et al. (2014a) | (1) Objective sleep parameters were measured using wrist actigraphy. (2) Subjective sleep quality was assessed using the PSQI. | Depressive symptoms were measured using the GDS. | - Self-administered questionnaire: (1) Demographics (birth date, ethnicity, years of education) (2) Smoking, alcohol consumption, caffeine intake, exercise habits  (3) Medical history/medication used  (4) BMI (5) Activity of daily living was measured by impairments in IADLs. (6) Cognition was measured by the MMSE. | Univariate comparisons of covariates across levels of baseline depressive symptoms categories were made using ANOVA or Kruskal Wallis tests for continuous variables and chi-squared homogeneity tests for categorical variables. | Objective assessments revealed an association between baseline prolonged wake after sleep onset (WASO ≥ 60 min) and worsening depressive symptoms at follow-up (OR = 1.36; 95% CI = 1.01-1.84; p = 0.046). There were no associations between other objectively assessed sleep measures and worsening depressive symptoms. | (1) It cannot generalize to other populations since our sample are community-dwelling women. (2) There is a limit ability to detect associations between sleep disturbances and depressive symptoms since the sample did not have more chronic or severe depressive symptoms.  (3) Conclusions about psychiatric diagnosis cannot be made because depressive symptoms were not assessed by a clinical diagnostic interview. (4) It cannot definitively determine whether the participant is sleeping or awake since gold standard of measurement was not utilized. (5) It relies on self-reported times in and out of bed, which may be inaccurate and could introduce errors into measurements. | N/A |
| Maglione et al. (2012) | (1) Objective sleep was measured by SleepWatch-O actigraph. Participants also completed sleep diaries for the time period they wore the actigraph. The diaries included information about times participants got into and out of bed and times. (2) Subjective sleep quality was assessed using the PSQI. (3) Subjective daytime sleepiness was measured using the ESS. | Depression was measured using the GDS. | Self-administered questionnaire: (1) Demographics (age, ethnicity, education was recorded at baseline) (2) Health status, smoking, alcohol consumption, caffeine intake, exercise, and medical history at visit 8 (3) Medications were recorded and categorized according to a computerized coding dictionary.  (4) Cognition was assessed using the MMSE. | Differences in the characteristics of the participants according to level of depressive symptoms were assessed using a chi-squared test for categorical variables, analysis of variance for normally distributed continuous data, and Kruskal-Wallis tests for skewed continuous data. Pearson's correlations were preformed to determine the relationship between subjective and objective sleep variables in the entire sample and in each subgroup of women by level of depressive symptoms. Objective and subjective sleep measures were analyzed as continuous variables using linear regression models and the least-squared means strategy was used to estimate the mean and 95% CI for each sleep parameter by level of depressive symptoms. Tests of linear trend were performed to detect graded associations. Associations between depressive symptoms and dichotomous sleep variables were assessed using logistic regression and a test for linear trend. | Baseline objectively measured WASO ≥ 60 min was associated with 1.5-fold increased odds having “some depressive symptoms” at follow-up in base models. However, this association was attenuated and no longer significant in multivariate models. There was a significant association between baseline WASO ≥ 60 min and 1.4-fold increased odds worsening depressive symptoms at follow-up in base models. This association remained significant and was only slightly attenuated in multivariate models. In contrast, there were no associations between other objectively measured sleep disturbances and either level of depressive symptom or increase in GDS score at follow-up. | (1) Hypothesis were not answered by the analysis and outcome measured were not predefined.  (2) It cannot generalize to other populations since our sample are community-dwelling women. (3) There is a limit ability to detect associations between sleep disturbances and depressive symptoms since the sample did not have more chronic or severe depressive symptoms.  (4) Conclusions about psychiatric diagnosis cannot be made because depressive symptoms were not assessed by a clinical diagnostic interview. (5) It cannot definitively determine whether the participant is sleeping or awake since gold standard of measurement was not utilized. | N/A |
| Palmius et al. (2017) | Global Positioning System (GPS) data was measured by an is a fusion of different location data sources via android | Depression was measured using the QIDS-SR16. | Self-administered questionnaire: demographics (gender, age, BMI and employment status) | Data preprocessing consisted of 3 steps; filtering, data down sampling, and data imputation [Missing data were imputed in sections where the participant was recorded at a location within 500 m either end of the missing section, and where the missing section had a length of 1) 2 h or less any time; or 2) 12 h or less after 9 PM. The missing section was filled with the mean latitude and longitude of the coordinates on either end. Extracting Location Clusters was done by (1) Extracting Stationary Locations (2) Clustering Stationary Locations Features were extracted from the preprocessed location data. Feature Calculation on Data Subsets were calculated as:  (1) Base Subset  (2) Weekday Subset  (3) Weekend Subset  (4) Median Subset (5) Optimized Daily Exclusion Subset.  The QIDS Score Estimation (questionnaire responses) and Depression Classification was done by two models. The first model is a standard linear regression, and the second model is a generalized linear model (GLM). | Healthy control participants did not report depressive symptoms and their features showed similar distributions to nondepressed Bipolar Depression participants. Questionnaire score estimation using geolocation-derived features from BD participants demonstrated an optimal mean absolute error rate of 3.73, while depression detection demonstrated an optimal (median ± interquartile range (IQR)) F1 score of 0.857 ± 0.022 using five features (classification accuracy: 0.849 ± 0.016; sensitivity: 0.839 ± 0.014; specificity: 0.872 ± 0.047). | (1) The performance of the method described is highly dependent on the data quality, which was found to be unpredictable on the Android platform used. (2) Geolocation recorded from smartphones only provides data as to the location of the handset and not necessarily its owner (3) Weekday and weekend patterns were used as proxies for working and nonworking days, which in many cases is likely to not be entirely accurate. (4) Self-reporting of mood symptoms also has a number of limitations given the inherent bias in retrospective recall of mood states (5) Only seven participants in the studied cohort exhibited depressive symptoms, and therefore, these results need to further validated on a larger scale. | It needs to explore mood changes within individuals but may prove to be useful tools in the early identification of depressive episodes and in guiding self-management. |
| Pye et al. (2021) | (1) Self-reported sleep quality was measured by the PSQI. (2) Sleep Regularity, Non-parametric patterns of activity, Cosinor (mean activity) was measured by the Actiwatch. | Depressive symptoms were measured via self-report using the 15-item version of the GDS-15. | (1) Medical history, medication use, and measured BMI was assessed by a semi-structured interview with clinicians. The presence of heart disease, hypertension, hypercholesterolemia, diabetes, and whether the participant ever smoked tobacco on a regular basis across their lifetime is recorded.  (2) The degree of medical burden was assessed using the Cumulative Illness Rating Scale-Geriatric (CIRS-G). (3) Alcohol intake was recorded by clinicians. (4) Global cognition was recorded using the MMSE to assess for dementia. | For non-parametric tests, analysis of variance, median and interquartile range were applied. For categorical data, chi-squared tests were used. All multiple comparisons were corrected, with alpha set at 0.05. Correlation coefficients used Pearson's or Spearman's rho in the case of non-normal distributions. We did not adjust alpha levels. All tests were two-tailed. MMSE scores, BMI, PSQI scores, and GDS-15 scores were included in the imputation model, with predictive mean matching used as the method. | (1) Older adults with current major depression had significantly less regular sleep.  (2) Cosinor and non-parametric measures of rest-activity patterns revealed significant differences between groups. Relative amplitude was significantly reduced in those with current MDD, and activity during post-midnight hours was significantly higher than controls, despite no difference in average sleep onset or offset times.  (3) There was a significant association between increased sleep regularity and a lower BMI in control (CON). Greater activity during the most active 10-hours was associated with a lower BMI in CON, and decreased medical burden in DEP-R. There were no significant associations between Mean estimation standard of rhythm (MESOR) and the included clinical variables in CON, DEP-R or DEP-C. | (1) It cannot reliably determine if there were significant differences in awakenings or sleep onset latency without PSG. (2) It reduces the ability to detect relationships of sleep regularity and rest-activity patterns with clinical correlates since our sample represents a restricted range and may not be representative of those with more severe symptoms. (3) Hypnotics may also influence activity patterns; however measurement using actigraphy is lacking for these drugs. | It should have larger samples, which may benefit from stratification of participants by use of these medications to determine any potential interactions with actigraphy measurement. |
| Gruenenfelder-Steiger et al. (2017) | (1) Daily physical activity was measured by accelerometer (movisens3; movisens GmbH, 2016).  (2) Daily Need-Fulfillment was measured with three items from the General Causality Orientations Scale. | Depression was measured by 3 items reflecting typical depressive mood from the Beck Depression Inventory (BDI). | Self-administered questionnaire: demographics (age and sex) | Multilevel modeling allows the analysis of such data structure appropriately, where multiple measurements are nested within a person. Therefore, we used multilevel analysis to investigate our research questions. We first examined the nested structure of the data by computing ICCs and compared how much of the total variance lied within-person. Second, we compared whether a random-intercept or a random-intercept and random-slope structure. | (1) It found a significant negative association between daily physical activity and daily depressive mood within-persons. That is, on days when participants engaged in more physical activity than usual, they reported less depressive mood. We also found that, on a given day with higher need-fulfillment, participants reported less depressive mood. (2) In models containing both physical activity and need-fulfillment as predictors of depressive mood, the effect of physical activity was attenuated to non-significance. (3) Daily need-fulfillment did not mediate the association between daily physical activity and daily depressive mood in older adults. However, the direct and total effects of daily physical activity were significant. | (1) The study period of 7 days may not have been long enough to get a valid picture of the everyday life of older people although we are convinced that a week of assessment did give us some idea about the associations between the examined processes (2) Participants response to self-report questions only at the end of each day.  (3) It uses only 3 items to assess need-fulfillment.  (4) In our multilevel models we looked only in one direction, that is, from physical activity toward depressive mood. However, it is also possible that lower depressive mood might lead to higher physical activity. | (1) We should assess a higher number of participants and cover a longer assessment period to provide more information about its associations over several weeks.  (2) We should investigate potential time-lagged effects and circadian patterns.  (3) We should use more items to capture the needs of autonomy, competence, and relatedness in greater detail.  (4) We should have a higher frequency of assessments per day. (5) We should test for other potential mechanisms underlying the within-person associations between physical activity and depressive mood in daily life |
| Maglione et al. (2014b) | Activity rhythms were measured by actigraph (SleepWatch-O, Ambulatory Monitoring, Inc., Ardsley, NY). | Depression was measured using the GDS. | (1) Self-administered questionnaire: Health status, smoking, alcohol consumption, caffeine intake, exercise habits, and medical history at SOF visit 8  (2) IADLs was measured by a clinic interview (3) Height and weight for BMI were measured by a physical examination  (4) Medications taken daily or almost daily during the prior 30 days were recorded and categorized according to a computerized coding dictionary.  (5) Cognition was assessed using the MMSE. | Differences in the characteristics of participants of depressive symptoms were assessed using a chi-square test for categorical variables and ANOVA for normally distributed continuous data. Circadian activity rhythm measures were expressed as continuous variables using linear regression models, and the least-squared means were used to estimate the mean (95% CI). Post-hoc analyses were performed using the Bonferroni adjustment. Logistic regression was used to determine the odds (95% CI) of being in the lowest quartile of circadian rhythm parameters by depression group. The normal group served as a referent group for these comparisons. Probability values for linear trend across the categories were calculated to see if a linear relationship existed. | Greater levels of depressive symptoms are associated with more desynchronization of circadian activity rhythms in community-dwelling older women. Specifically, in multivariate models, greater levels of depressive symptoms were associated with decreased robustness of circadian activity rhythms. Greater levels of depressive symptoms were also associated with decreased amplitude. We also found a graded association between greater levels of depressive symptoms and an earlier evening settling time in multivariate models. | (1) It is not possible to determine whether circadian activity rhythm disturbances are a risk factor for depression because this study is cross-sectional,  (2) It cannot generalize to other population since the sample is made up of community-dwelling women. (3) It does not provide information about differences in stages of sleep or REM latency via actigraphy. (4) It is not able to look at associations with specific diagnoses because our assessment of depression was not based on a diagnostic interview. | We should conduct longitudinal analyses to further explore the relationship between depressive symptoms and circadian rhythm disturbances. |
| Paudel et al. (2013) | (1) Objective sleep/wake parameters were measured by passive sensing. (2) Subjective sleep quality and sleep disturbances over the past month were measured by the PSQI and the ESS. (3) The duration participants wore the actigraph (time into, time out, time when the actigraph was removed) was measured by sleep diaries. | Depressive symptoms at baseline and follow-up were measured by the 15-item GDS. | Self-administered questionnaire: (1) Medication used within the past 30 days. (2) A history of physician-diagnosed stroke, parkinsonism, diabetes mellitus, chronic obstructive lung disease, congestive heart failure, chronic kidney disease, and myocardial infarction  (3) Cognitive function was assessed using the MMSE (4) Body weight was measured using a balance beam or digital scale. Height was measured using a wall-mounted stadiometer.  (5) Health status, alcohol intake, caffeine consumption, smoking status, walking for exercise, and IADL were interviewed by a trained technician. | Depression status at the follow-up examination were compared using analysis of variance for normally distributed continuous data, Kruskal-Wallis for skewed continuous data, and chi-square tests for categorical data. Associations between sleep disturbances at baseline and odds of being depressed at follow-up were examined using logistic regression in the entire Cohort. Models included an age- and clinic site-adjusted base model, the base model further adjusted for number of baselines GDS symptoms, and the base model that further adjusted for multiple potential confounders. | (1) Poor self-reported sleep quality at baseline was independently associated with greater odds of incident depression at follow-up. (2) Those with poor sleep efficiency, prolonged sleep latency, greater nighttime wakefulness, multiple long-wake episodes, and greater burden of objectively measured sleep/wake disturbances also had greater odds of being depressed at the follow-up. (3) There is no association of objectively assessed TST or reports of daytime sleepiness with depression status at follow-up. | (1) Results may not apply to other populations because this cohort included only community-dwelling Caucasian men.  (2) It relies on a self-reported questionnaire to assess depressive symptoms and depression. Although this scale has performed well in validation studies, it is not a clinical measure of depression.  (3) Information on history of depression prior to baseline has not been assessed. (4) Although these men are generally healthy at baseline, there remains the question about how sensitive the GDS is to change in symptoms in older men.  (5) The follow-up time is short and some of the associations may reflect reverse causality. | (1) We should further evaluate the sensitivity of the GDS to change and confirm results using other measures of depression. (2) We should examine longitudinal associations using alternative objective parameters and explore the interrelationships between objective and subjective measures of sleep. |
| Abbas et al. (2022) | ActiGraph used a unit of measurement called ‘count’ in relation to activity measurements. | The severity of apathy was measured by the Apathy Evaluation Scale (AES). | N/A | Counts are the result of summing post-filtered acceleration values around each axis into epochs, as indicated in raw data being sampled at 30 Hz. Data were segmented using 1-hour segments. Consequently, a M × N × 3 matrix resulted from previous operations. The time-series were analyzed to extract some parameters that might reveal the signs of apathy in terms of physical motion. Once the pre-processing was done, we proceeded to feature extraction, leading to six features. Afterwards, the step ahead prediction is computed using the second half of the window. Feature 6 or the goal here is to fit an Auto Regressive Moving-Average model is the mean of the residuals, which are the difference between real and the predicted data. | Our pilot study showed that motion signals, measured by a wrist-worn sensor, might provide a good indicator of apathy. The proposed features were able to separate apathetic individuals from other subjects with a quite acceptable accuracy, after undergoing a non-linear transformation using the sigmoidal function and an orthogonal transformation by Principle Component Analysis (PCA), converting features into uncorrelated variables. We also found with these preliminary results that the linear regression model outperformed other models when it comes to the prediction of AES scores. | (1) The study cohort being relatively small. (2) The analyzed data are counts, which are the result of summing post-filtered acceleration measures. This preprocessing is valuable to remove unwanted components before the analysis, but might also exclude some hidden information from raw acceleration data which may increase the discrimination power of the system | Identification of long inactive periods and recurrent sedentary lifestyle is going to be an objective of a future work. |
| Lee et al. (2014) | Daily step counts were recorded with the accelerometer Actigraph AM-7164. | Depressive symptoms were measured by the Patient Health Questionnaire (PHQ-9). | Self-administered questionnaire: (1) Demographics (age, gender, race/ethnicity, marital status, education, annual household income) (2) Chronic medical conditions and medication (3) BMI (4) Smoking status and alcohol use in the past 12 months | Binary logistic regression analyses were used to assess predictors of moderate depressive symptoms, including physical activity and demographic variables. Next, a similar series of bivariate logistic regressions examined predictors of sedentary physical activity. Variables that were found to be significantly associated with depressive symptoms and/or physical activity at the bivariate level were retained for use in multivariate logistic regression models. Results were reported as OR with a 95% CI. | (1) There is a significant relationship between physical activity and depressive symptoms, even when controlling for other factors. (2) Age and chronic medical conditions were also significantly predictive of depressive symptoms in both bivariate and multivariate analyses. Low income was also found to be related to increased depressive symptoms, although only in the bivariate analyses. | (1) More sedentary individuals may have been screened out due to incomplete physical activity data because those who are already more highly active are more inclined to wear the accelerometer than those who are less active. Thus, the percentage of active individuals may be exaggerated.  (2) Individuals with depression may be underrepresented in the study because their mood may be associated with poor participation.  (3) Generalizability of the results is limited. The data include only community-dwelling populations and conformed to a cross-sectional design. (4) Limited factors are included to explain depression as the number of individuals exhibiting depression was too low to support many predictors in the model. | We should analyze the observed relationships among physical activity, depression, and selected demographic variables longitudinally and in larger samples |
| Smagula et al. (2015b) | Sleep/wake activity (rhythm height, timing, and robustness) were measured by actigraphy. | Depressive symptoms were measured using the GDS. | Self-administred questionnaire: (1) Demographics (age, study site, race, educational attainment) (2) Smoking, alcohol consumption, caffeine intake, physical activity  (3) Medical history/medication used such as osteoarthritis, hypertension and stroke (4) Anxiety was measure by the Goldberg Depression and Anxiety Scale (GDAS) and psychosocial factors such as stress. (5) BMI (6) Activity of daily living was measured by IADLs and falling.  (7) Cognition was measured by the Modified Mini-Mental State (3MS). (8) Objective sleep was measured by Actigraph and PSG while subjective sleep was measured by PSQI. | Latent class analysis was applied to this study. The number of latent groups was selected based on both interpretability and model fit statistics. The Bayesian Information Criterion (BIC) was used to compare models with a different number of latent classes (sub-groups).  Growth curve modeling was implemented using SAS PROC MIXED with and random slopes and intercepts, with time expressed as a continuous variable (in years from baseline). First, separate base models (adjusted for age and study site) assessed crude associations between latent activity rhythm sub-groups and the level or rate of change in the outcome over time (associations with the rate of change were examined as the interactions between activity rhythm sub-group and time). Associations between all covariates and the level or rate of change in the outcome were similarly assessed. From these separate models, a maximum multivariable was constructed including all associations that achieved at least p<0.10 with the level (intercept) or rate of change (slope) in depression symptoms over time. To achieve a parsimonious final model, variables which were not significantly associated with the outcome in the maximum model (p<0.10) were removed. | (1) We found eight sub-groups with distinct combinations of activity rhythm disturbances: one had all normative activity rhythm parameters (32.09%), one had only lower activity (10.06%), three had earlier activity (totaling 26.96%), and three had later activity (totaling 30.89%). (2) We next examined whether the derived activity rhythm sub-groups were associated with different rates of change in depression symptom levels over an average of 5.5 (0.52 SD) follow-up years. The sub-group with lower activity only had faster increases in depressive symptoms over time (compared to the group with normative rhythm parameters), but this association was accounted for by adjustments for concurrently assessed health status covariates. Independent of these covariates, we found four activity rhythm disturbance sub-groups that experienced faster depressive symptom increases (compared with the normative sub-group): These included all three sub-groups that had later activity timing, and one sub-group that had earlier activity timing plus a shorter active period and a dampened rhythm. | (1) Actigraph-recorded activity rhythms reflect the activity of the master biological keeper in the suprachiasmic nucleus but should not be interpreted as a direct indicator of circadian biology. (2) The MrOS Sleep Study consists of older men who were mostly white, and these findings cannot necessarily generalize to other populations. The derivation of sub-groups via Latent Class Analysis (LCA) and subsequent use of these sub-groups to predict change in a longitudinal mixed model introduces measurement error.  (3) Residual confounding may have influenced our results, in particular, our analysis did not account for a potential role of past depressive episodes in relation to current activity rhythms and future depression symptoms. (4) The average magnitude of depression severity and changes in severity associated with these activity rhythm groups was not large. | Future research is also needed determine how different combinations activity rhythm disturbances might synergistically affect depression and other aspects of health. |

Note: Independent Variables (IV); Dependent Variables (DV); Confounding Variables (CV)
