# Supplementary material for: Depressive symptom screening in elderly by passive sensing data of smartphones or smartwatches: A systematic review
Source: PLoS One. 2024 Jun 27;19(6):e0304845. doi: 10.1371/journal.pone.0304845 (PMC11210876; doi:10.1371/journal.pone.0304845)
Supplement: S4 Table — (DOCX) [file pone.0304845.s005.docx]

**S4 Table** Summary of reviewed studies’ quality assessment

| Study design | JBI - Risk of Bias | Choi et al. (2022) | Smagula et al. (2015a) | Vesel et al. (2020) | Kim et al.(2019) | Aubourg et al. (2019) | O'Brien et al. (2016) | Maglione et al. (2014a) | Palmius et al. (2017) | Gruenenfelder-Steiger et al. (2017) | Abbas et al. (2022) | Paudel et al. (2013) | Smagula et al. (2015b) |
| --- | --- | --- | --- | --- | --- | --- | --- | --- | --- | --- | --- | --- | --- |
| Cohort | 1. Were the two groups similar and recruited from the same population? | Low | Low | Low | Low | Unclear | Low | Low | Low | Low | Low | Low | Low |
|  | 2. Were the exposures measured similarly to assign people to both exposed and unexposed groups? | Low | Low | Low | Low | Low | Low | Low | Low | Low | Low | Low | Low |
|  | 3. Was the exposure measured in a valid and reliable way? | Low | Low | Low | Low | Low | Unclear | Low | Low | Low | Low | Low | Low |
|  | 4. Were confounding factors identified? | High | Low | Low | High | High | Low | Low | Low | Low | Unclear | Low | Low |
|  | 5. Were strategies to deal with confounding factors stated? | High | Low | High | High | High | Low | Low | Unclear | Low | Unclear | Unclear | Low |
|  | 6. Were the groups/participants free of the outcome at the start of the study (or at the moment of exposure)? | High | Unclear | Unclear | Low | Unclear | Low | Low | High | Low | High | Low | Unclear |
|  | 7. Were the outcomes measured in a valid and reliable way? | Low | Low | Low | Low | Low | Low | Low | Low | Low | Low | Low | Low |
|  | 8. Was the follow up time reported and sufficient to be long enough for outcomes to occur? | Low | Low | Low | Low | Low | High | Low | Low | High | High | Low | Low |
|  | 9. Was follow up complete, and if not, were the reasons to loss to follow up described and explored? | Low | Low | Unclear | Low | Unclear | Low | High | Unclear | High | Unclear | Low | Low |
|  | 10. Were strategies to address incomplete follow up utilized? | Unclear | Unclear | Unclear | Unclear | Unclear | Low | High | Unclear | High | Unclear | Low | Unclear |
|  | 11. Was appropriate statistical analysis used? | High | Low | High | High | High | Low | Low | Low | Low | Low | Low | Low |

| Study design | JBI - Risk of Bias | Cabanas-Sánchez et H al. (2021) | Alcántara et al. (2016) | Luik et al. (2015) | Maglione et al. (2012) | Maglione et al. (2014b) | Lee et al.(2014) | Asai et al.(2018) |
| --- | --- | --- | --- | --- | --- | --- | --- | --- |
| Cross-sectional | 1. Were the criteria for inclusion in the sample clearly defined? | Low | Low | Unclear | Low | Low | Low | Unclear |
|  | 2. Were the study subjects and the setting described in detail? | Low | Low | Low | Low | Low | Low | Low |
|  | 3. Was the exposure measured in a valid and reliable way? | Low | Low | Low | Low | Low | Low | Low |
|  | 4. Were objective, standard criteria used for measurement of the condition? | NA | NA | NA | NA | NA | NA | NA |
|  | 5. Were confounding factors identified? | Low | Low | Low | Low | Low | Low | Low |
|  | 6. Were strategies to deal with confounding factors stated? | Low | Low | Low | Low | Low | Low | Low |
|  | 7. Were the outcomes measured in a valid and reliable way? | Low | Low | Low | Low | Low | Low | Low |
|  | 8. Was appropriate statistical analysis used? | Low | Low | Low | Low | Low | Low | Low |

| Study design | JBI - Risk of Bias | Pye et al. (2021) | Hoyos et al. (2020) |
| --- | --- | --- | --- |
| Case-control | 1. Were the groups comparable other than the presence of disease in cases or the absence of disease in controls? | Low | Low |
|  | 2. Were cases and controls matched appropriately? | Low | Low |
|  | 3. Were the same criteria used for identification of cases and controls? | Low | Low |
|  | 4. Was exposure measured in a standard, valid and reliable way? | Low | Low |
|  | 5. Was exposure measured in the same way for cases and controls? | Low | Low |
|  | 6. Were confounding factors identified? | Low | Low |
|  | 7. Were strategies to deal with confounding factors stated? | Low | Unclear |
|  | 8. Were outcomes assessed in a standard, valid and reliable way for cases and controls? | Low | Low |
|  | 9. Was the exposure period of interest long enough to be meaningful? | Low | Low |
|  | 10. Was appropriate statistical analysis used? | Low | Low |
